# Bioenergetic costs and the evolution of noise regulation by microRNAs

**DOI:** 10.1073/pnas.2308796121

**Published:** 2024-02-22

**Authors:** Efe Ilker, Michael Hinczewski

**Affiliations:** ^a^Max Planck Institute for the Physics of Complex Systems, Dresden 01187, Germany; ^b^Department of Physics, Case Western Reserve University, Cleveland, OH 44106

**Keywords:** microRNAs, gene regulatory networks, evolution, bioenergetics

## Abstract

Noise control, together with other regulatory functions facilitated by microRNAs (miRNAs), is believed to have played important roles in the evolution of multicellular eukaryotic organisms. miRNAs can dampen protein fluctuations via enhanced degradation of messenger RNA (mRNA), but this requires compensation by increased mRNA transcription to maintain the same expression levels. The overall mechanism is metabolically expensive, leading to questions about how it might have evolved in the first place. We develop a stochastic model of miRNA noise regulation, coupled with a detailed analysis of the associated metabolic costs. Additionally, we calculate binding free energies for a range of miRNA seeds, the short sequences which govern target recognition. We argue that natural selection may have fine-tuned the Michaelis–Menten constant $K_{M}$ describing miRNA–mRNA affinity and show supporting evidence from analysis of experimental data. $K_{M}$ is constrained by seed length, and optimal noise control (minimum protein variance at a given energy cost) is achievable for seeds of 6 to 7 nucleotides in length, the most commonly observed types. Moreover, at optimality, the degree of noise reduction approaches the theoretical bound set by the Wiener–Kolmogorov linear filter. The results illustrate how selective pressure toward energy efficiency has potentially shaped a crucial regulatory pathway in eukaryotes.

## Significance

MicroRNAs are short strands of genetic material that regulate cellular functions, including reducing noise in protein numbers. We argue that this regulation incurs a steep energetic price so that natural selection drives such systems toward greater energy efficiency. This involves tuning the interaction strength between microRNAs and their target messenger RNAs, which is controlled by the length of a microRNA seed region that pairs with a complementary region on the target. We show that 6 to 7 nucleotide seed lengths are optimal, which may help explain why seeds of this size are prevalent in animal microRNAs. Moreover, the behavior of the optimal microRNA network mimics the best possible linear noise filter, a classic concept in engineered communications systems.

Nonequilibrium processes within living systems exact a high price: the constant maintenance of fuel molecules and raw materials at sufficient concentrations to provide thermodynamic driving potentials for biological function (1). Optimizing that function with respect to thermodynamic costs is a factor constraining evolution, and would have been particularly important at the very earliest stages of life, where the metabolic chemistry responsible for maintaining those potentials was necessarily primitive and relatively inefficient. Yet thermodynamic costs are not the only factor that matters, and biology is full of counter-intuitively complex chemical mechanisms whose evolutionary predecessors, perhaps arising out of the randomness of genetic drift, may have consumed energy resources without any clear fitness benefit.

The discovery of microRNAs (miRNAs) along with their counterparts, small noncoding RNAs, raised many open questions about their functional purposes and evolution (2, 3). These short endogenous RNAs, around 22 nucleotides (nt) in length, exist in many eukaryotic cells. They constitute the core of the RNA-induced silencing complex (RISC) that interacts with target messenger RNA (mRNA), leading to translational repression and the accelerated degradation of their target by a mechanism known as the RNA interference. One possible functional role for this interference, which is the focus of our work, is fine-tuning noise in protein populations by reducing the variance of protein copy numbers (4, 5), conferring robustness to cellular functions (6). Such noise control, together with other regulatory functions facilitated by microRNAs, is believed to have played important roles in the evolution of complex multi-cellular life (7–10). Yet it is a considerable expenditure of resources, similar to setting up a factory production line for a valuable good, funding gangs of thieves to constantly raid the factory, and compensating for losses by increasing the production rate. So how would such a regulation scheme arise, and has evolution actually optimized it? Using a combination of statistical physics, information theory, biochemistry, and population genetics, we arrive at some tentative answers to these questions.

Our results give insights into one peculiar feature of this system: why the job requires such short RNA molecules—the significance of the “micro” in microRNA. Specific interactions between the miRNA and its target in fact largely depend on only a 6- to 8-nt sequence known as the miRNA seed region, which forms Watson–Crick base pairs with a complementary sequence on the target mRNA. We argue that the observed length of the seed region lies in a metabolic sweet spot, giving just enough affinity between the miRNA and mRNA (measured by their Michaelis–Menten constant $K_{M}$) to optimally control noise at a given level of energetic expenditure. Longer seed sequences (higher affinity, smaller $K_{M}$) would increase rather than decrease noise. On the other hand, shorter seed sequences (lower affinity, larger $K_{M}$), while allowing interactions with a wider range of targets, would also require significantly more energy to achieve the same level of control. By estimating these energy expenditures, we also show that effective noise control is costly enough to be under selection pressure in eukaryotic cells. A novel miRNA may initially appear during the course of evolution as a random product of genetic drift with nonoptimal parameters and then get gradually repurposed as a noise control mechanism that confers a fitness advantage. Once this starts to occur, our theory predicts that natural selection would hone $K_{M}$ toward an optimal value.

## miRNA Regulation as a Noise Filter for Protein Expression.

**Fig. 1.**
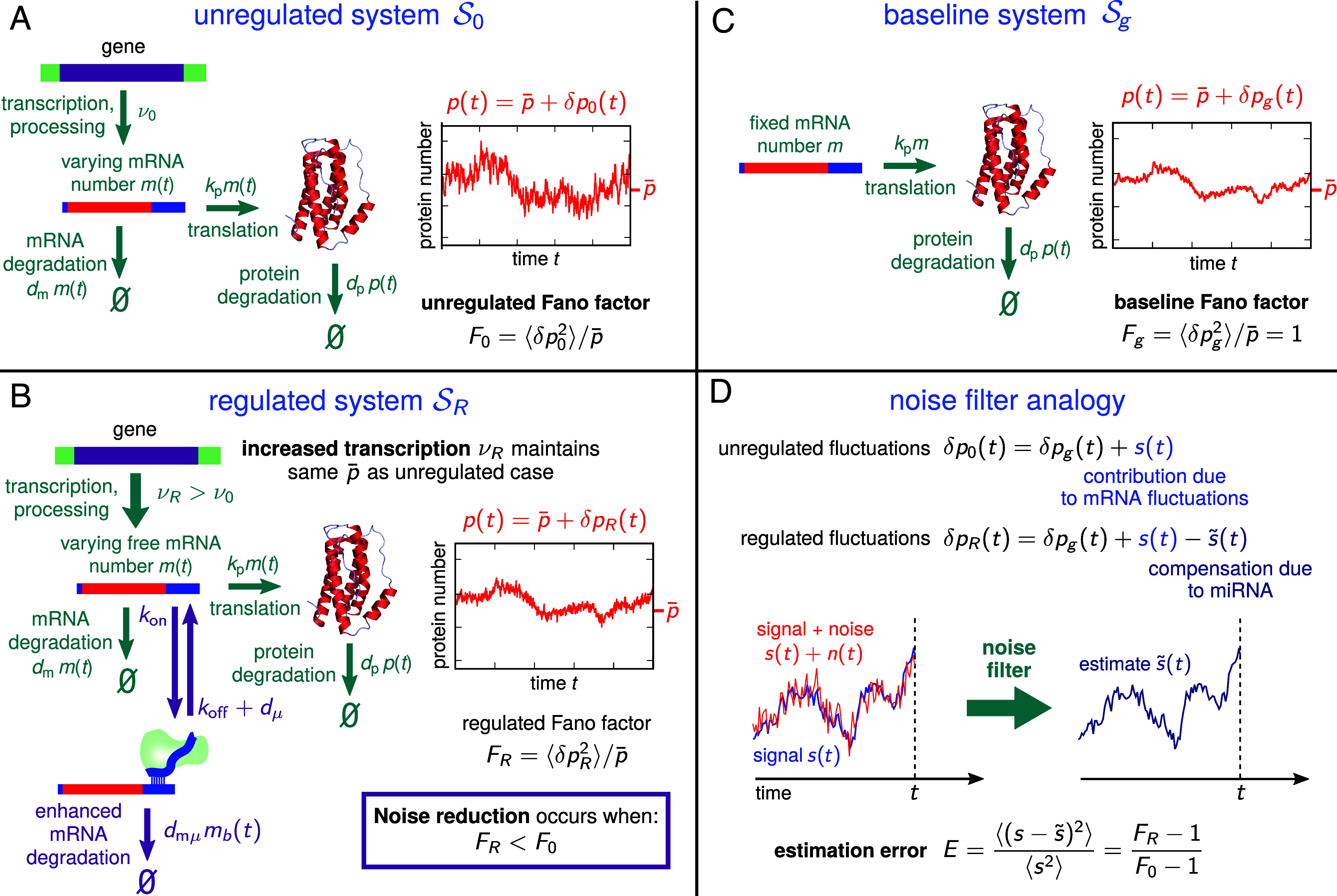
Overview of the miRNA noise regulation model: (*A*) In the unregulated system $S_{0}$ with no miRNA, noise in protein numbers has contributions from both varying mRNA population and intrinsic noise in the translation process, leading to a Fano factor $F_{0}$ (protein variance divided by the mean protein number $\overset{¯}{p}$). (*B*) For the regulated system $S_{R}$, enhanced mRNA degradation due to targeting by miRNA can reduce protein noise, leading to a Fano factor $F_{R}<F_{0}$. To compensate for the loss of mRNA and achieve the same $\overset{¯}{p}$, the transcription rate $\nu_{R}$ must be increased relative to its value $\nu_{0}$ in $S_{0}$. (*C*) To set up the noise filter analogy, we introduce an imaginary baseline system $S_{g}$, which has fixed mRNA population and hence the protein noise is solely due to translation. The resulting Fano factor $F_{g}=1$ is a lower bound on the two systems above: $1\leq F_{0}$, $F_{R}$. (*D*) Decomposing the unregulated/regulated protein fluctuations $\delta p_{0}\left(t\right)$ and $\delta p_{R}\left(t\right)$ into a baseline contribution $\delta p_{g}\left(t\right)$ and an additional contribution allows us to define the signal s(t) and estimate $\tilde{s}\left(t\right)$ in the noise filter analogy. Their normalized mean-squared difference E is the estimation error of the filter, which can be expressed in terms of $F_{R}$ and $F_{0}$.

We start our theoretical description by introducing the architecture of the miRNA-regulated system $S_{R}$ (Fig. 1*B*) in comparison to the unregulated one $S_{0}$ (Fig. 1*A*). Our description builds off the experimentally validated model of ref. 5 and takes the form of stochastic biochemical reaction networks with dynamics obeying linearized chemical Langevin (CL) equations (11, 12), as detailed in *SI Appendix*, section S.I A. The CL results agree closely with available experimental data (5) and give us analytical expressions for correlation functions describing copy number fluctuations of chemical species in each system.

The fluctuating output numbers are denoted as $x(t)=\overset{¯}{x}+\delta x(t)$ where δx(t) is the variation from the mean level $\overset{¯}{x}$. The main species of interest are total (bound + unbound) miRNA, mRNA, and protein copy numbers, denoted by $x=\mu_{\mathrm{tot}}$, m, or p respectively. In the unregulated system $S_{0}$ (Fig. 1*A*), we have transcription from a gene at rate $\nu_{0}$ producing an mRNA population m(t), which is then translated with rate constant $k_{p}$ to a produce a protein population p(t). The mRNA and proteins are degraded with rate constants $d_{m}$ and $d_{p}$, respectively.

To meaningfully compare $S_{R}$ to $S_{0}$, we assume parameter sets such that the mean protein output $\overset{¯}{p}$ is the same for both regulated and unregulated systems. This maintains the functional effectiveness of the protein in the regulated system, but with the potential added benefit of noise reduction. Indeed, miRNA are often up-regulated along with their target mRNA via feedforward loops (13, 14). For $S_{R}$ (Fig. 1*B*), there is an RNA interference mechanism: free miRNAs with population μ(t) can bind to the mRNA with rate constant $k_{\mathrm{on}}$ to form a bound complex with population $m_{b}\left(t\right)$. Considering a single miRNA binding site on the target mRNA, the total number of miRNA is $\mu_{\mathrm{tot}}\left(t\right)=\mu \left(t\right)+m_{b}\left(t\right)$. The mRNA in the complex has an enhanced degradation rate constant $d_{m\mu }>d_{m}$ relative to the regular mRNA value $d_{m}$. The miRNA unbinds with rate constant $k_{\mathrm{off}}$ and degrades with $d_{\mu }$. For simplicity, we assume the miRNA degradation rate constant $d_{\mu }$ is the same when both bound and unbound to mRNA (5). miRNA–mRNA affinity can be characterized through the Michaelis–Menten constant,

[1] $$\begin{array}{c} K_{M}=K_{D}+k_{\mathrm{cat}}/k_{\mathrm{on}}, \end{array}$$

where the dissociation constant $K_{D}=k_{\mathrm{off}}/k_{\mathrm{on}}$ and $k_{\mathrm{cat}}=d_{m\mu }+d_{\mu }$ is an effective catalytic rate constant for the miRNA-catalyzed degradation reaction. $K_{M}$ approximately relates $m_{b}\left(t\right)$ to $\mu_{\mathrm{tot}}\left(t\right)$ through $m_{b}\left(t\right)\approx m\left(t\right)\mu_{\mathrm{tot}}\left(t\right)/\left(K_{M}+m\left(t\right)\right)$. Note that experimental values of $K_{M}$ and $K_{D}$ are reported in units of concentration (molars) while our CL formalism uses copy numbers of chemical species. We assume a typical eukaryotic cell volume V = 2,000 μm^3^ to convert between concentrations and copy numbers as needed.

Because of interference from the miRNA, the transcription rate $\nu_{R}$ of mRNA in $S_{R}$ must be larger than the rate $\nu_{0}$ in $S_{0}$, if both systems maintain the same mean protein level $\overset{¯}{p}$. In the limit of no miRNA ($\mu_{\mathrm{tot}}\to 0$) or vanishing affinity ($K_{M}\to \infty$), the regulated system approaches the same behavior as the unregulated one, with $\nu_{R}\to \nu_{0}$. The strength of regulation can be characterized by a parameter $R\equiv 1-\nu_{0}/\nu_{R}$, where R ranges between 0 (no regulation) to 1 (maximum possible regulation). For known miRNA-mediated regulation networks, R typically lies between 0.05 and 0.95 (15, 16).

To quantify the effect of miRNA on noise, we look at the Fano factor $F_{\lambda }=\left\langle {\left(\delta p_{\lambda }\right)}^{2}\right\rangle /\overset{¯}{p}$ with λ=R,0 labeling the system $S_{\lambda }$ in which the quantity is calculated. Successful noise reduction implies that $F_{R}<F_{0}$: for the same mean protein output level, there is less protein variance in the presence of miRNA. Qualitatively, this arises because the miRNA system reduces the number of translated proteins per mRNA, on average by a factor of 1−R, and hence decreases the susceptibility of translation to fluctuations in mRNA levels. When miRNA regulation is compensated for by transcriptional increase, it is thus possible to mitigate the propagation of noise from mRNA to protein numbers. However, there is a trade-off, because the stochasticity of miRNA–mRNA interactions, as well as fluctuating miRNA populations, also introduces noise into mRNA levels. This added noise can cancel out the protein noise reduction benefit in certain parameter regimes, for example, when miRNA–mRNA affinities are high.

To understand the role of miRNA more precisely, it is helpful to use a noise filter analogy. In order to motivate this mathematical analogy, we define an imaginary baseline system $S_{g}$ (Fig. 1*C*), where we have fixed the mRNA population at a constant level $m\left(t\right)=\overset{¯}{m}=\left(d_{p}/k_{p}\right)\overset{¯}{p}$, which agrees with the mean $\overset{¯}{m}$ in $S_{R}$ and $S_{0}$ and hence gives the same $\overset{¯}{p}$. This removes the contribution of mRNA fluctuations to the noise, so $p\left(t\right)=\overset{¯}{p}+\delta p_{g}\left(t\right)$, where $\delta p_{g}\left(t\right)$ are the ground-level (baseline) fluctuations that come from protein translation and degradation (and cannot be mitigated by RNA interference). As summarized in Fig. 1*D*, the protein fluctuations in both $S_{0}$ and $S_{R}$ can then be compared to this baseline. For $S_{0}$, we write $\delta p_{0}\left(t\right)=\delta p_{g}\left(t\right)+s\left(t\right)$, where s(t) represents the added noise due to varying mRNA population m(t). For $S_{R}$, this added noise is partially compensated, $\delta p_{R}\left(t\right)=\delta p_{g}\left(t\right)+s\left(t\right)-\tilde{s}\left(t\right)$. Both s(t) and $\tilde{s}\left(t\right)$ can be explicitly calculated using the CL formalism (*SI Appendix*, section S.II), and it turns out they are correlated: $\tilde{s}\left(t\right)$ takes the form of a convolution,

[2] $$\begin{array}{c} \tilde{s}\left(t\right)=\int_{-\infty }^{t}dt^{\prime }H\left(t-t^{\prime }\right)\left(s\left(t^{\prime }\right)+n\left(t^{\prime }\right)\right), \end{array}$$

where H(t) and n(t) are functions of the biochemical parameters. The above equation has a clear noise filter interpretation (depicted schematically in Fig. 1*D*): s(t) is the “signal,” n(t) a “noise” that corrupts the signal, H(t) is a linear filter function that acts via convolution on the past history of the corrupted signal s(t)+n(t), and $\tilde{s}\left(t\right)$ is the “estimate” of the signal. It turns out the problem of reducing protein noise via mRNA interference (making $F_{R}$ as small as possible) is equivalent to making $\tilde{s}\left(t\right)$ as close as possible to s(t). We can see this directly in the error of estimation, which is defined as $E=\left\langle {\left(s-\tilde{s}\right)}^{2}\right\rangle /\left\langle s^{2}\right\rangle$. For our case, E can be expressed in terms of the Fano factors, $E=\left(F_{R}-1\right)/\left(F_{0}-1\right)$. Fine-tuning miRNA parameters only affects $F_{R}$, leaving $F_{0}$ fixed, so E can be minimized by decreasing $F_{R}$. Both $F_{R}$ and $F_{0}$ are bounded from below by the Fano factor $F_{g}=1$ of the baseline system, so perfect filtering, E→0, would correspond to $F_{R}\to 1$. The noise filter interpretation, which has been earlier applied to a variety of other biological networks (for a review see ref. 17), has an important payoff which we will return to later: it allows us to find the conditions for optimal noise reduction and calculate tighter bounds on $F_{R}$, which in general will be greater than 1 at optimality.

## Bioenergetic Costs of miRNA Regulation.

The second major component of our model is an estimate of the costs for miRNA regulation, which we adapt from experimental data on eukaryotic transcription energetics collected in ref. 18. In general, this includes energy expenditures channeled to the synthesis of new molecules as well as maintenance, the recycling/repair of molecules to maintain steady-state levels (i.e., assembling mRNA from existing nucleotides to counterbalance degradation). Eukaryotic cells typically have long enough generation (cell division) times $t_{r}$, that maintenance is the dominant contribution to metabolic expenditures over a generation. Focusing on the maintenance costs, we can estimate the transcriptional metabolic consumption $C_{\nu }$ (in units of phosphate [P] bonds hydrolyzed, namely ATP or ATP-equivalents) for the unregulated system per generation: $C_{\nu }≃t_{r}M_{\nu }$, where $M_{\nu }=\nu_{0}\epsilon_{m}$ is the consumption rate. Here, $\nu_{0}$ is the mRNA transcription rate in $S_{0}$, and $\epsilon_{m}$ is the energy cost in terms of P for assembling the mRNA (which will depend on the length of the transcript). For the regulated case, there is an extra contribution $\delta C_{\nu }=t_{r}\Delta M_{\nu }$. The difference in consumption rate $\Delta M_{\nu }=\left(\nu_{R}-\nu_{0}\right)\epsilon_{m}+\nu_{\mu }\epsilon_{\mu }$. The first term accounts for the added costs of increased transcription to maintain the same $\overset{¯}{p}$, while the second term is the rate of miRNA assembly $\nu_{\mu }$ times the cost $\epsilon_{\mu }$ of that assembly in units of P (including potentially any related costs of the RISC complex). As shown in *SI Appendix*, section S.I C, $\Delta M_{\nu }$ can be expressed in terms of the biochemical parameters of the system as:

[3] $$\begin{array}{c} \Delta M_{\nu }=\frac{R}{1-R}\left(1+\gamma_{\mu }\left(1+\frac{K_{M}}{\overset{¯}{m}}\right)\sigma_{\epsilon }\right)M_{\nu }, \end{array}$$

with $\gamma_{\mu }\equiv d_{\mu }/d_{m\mu }$ and $\sigma_{\epsilon }\equiv \epsilon_{\mu }/\epsilon_{m}$. Based on experimental estimates (summarized in *SI Appendix*, Table S1), we know that $\gamma_{\mu }\ll 1$ and $\sigma_{\epsilon }\ll 1$. Given the experimental range of R = 0.05 to 0.95, the term R/(1−R) can vary by a factor of 361 between the smallest and largest observed regulation magnitudes, highlighting the strong dependence of $\Delta M_{\nu }$ on R. The remaining terms in Eq. **3** encapsulate the modification to the costs due the parameters governing miRNA–mRNA interactions, particularly $K_{M}$ and the degradation enhancement $d_{m\mu }$.

As discussed later in the section on evolutionary pressure, it is convenient to define a nondimensional measure for the extra cost due to regulation: the extra cost as a fraction of the total metabolic expenditure of a cell per generation, $\delta C_{T}/C_{T}$. Here $\delta C_{T}=\delta C_{\nu }$ and $C_{T}≃t_{r}M_{\text{tot}}$, where $M_{\text{tot}}$ is the total maintenance ATP consumption rate. Based on the data from ref. 18, $M_{\text{tot}}$ approximately scales with cell volume, and we use a value $M_{\text{tot}}=3\times 10^{11}$ P/hr characteristic of eukaryotic cells. Thus, we will report costs in terms of $\delta C_{T}/C_{T}=\Delta M_{\nu }/M_{\text{tot}}$, with $\Delta M_{\nu }$ given by Eq. **3**.

Our model so far has assumed one mRNA target, but in general, a single miRNA can target up to hundreds of mRNAs, which will also change the energetic costs of regulation. As a rough estimate of this multi-target scenario, we can assume similar biochemical parameters among different targets. This allows us to use the single-target theory but scaling up $\overset{¯}{m}$ (and hence $\overset{¯}{p}$) to reflect the total mRNA numbers when accounting for all targets. Note that the dependence of $\Delta M_{\nu }$ on $\overset{¯}{m}$ is nontrivial, since both R and $M_{\nu }$ depend on $\overset{¯}{m}$. However, when we demand a certain level of overall noise control (a specific value of E), the extra cost $\Delta M_{\nu }$ will increase with $\overset{¯}{m}$ as the number of targets gets larger: the larger the system, the more expensive it is to control.

## Seeds of Length 6 to 7 nt Are Most Energetically Efficient for Noise Reduction.

With all the components of the model defined, we can now investigate the interplay between noise reduction and energetic costs. The error E (or equivalently the Fano factor $F_{R}$) can be expressed as a function of $\Delta M_{\nu }$, $\gamma_{\mu }$, $\sigma_{\epsilon }$, $\overset{¯}{m}$ and $K_{M}$ (details in *SI Appendix*, sections S.I B and C). For a given cost $\Delta M_{\nu }$ and fixed degradation/energy parameters $\gamma_{\mu }$ and $\sigma_{\epsilon }$, we can ask what value of $K_{M}$ minimizes E. $K_{M}$ is an interesting tuning parameter because it is related to the binding strength between the miRNA and the mRNA, which in turn depends on the number of complementary interactions between the seed and the target region of the mRNA. From Eq. **1**, $K_{M}\geq K_{D}$, and the dissociation constant $K_{D}$ is related to the free energy of binding via $\Delta G^{0}=k_{B}T\ln\left(K_{D}/\left[1M\right]\right)$. Longer seeds should allow for more negative $\Delta G^{0}$ (stronger binding) and hence smaller values of $K_{D}$. This in turn gives access to smaller values of $K_{M}$. For RNA interference systems, experimentally measured $K_{M}$ values have ranged from comparable to $K_{D}$ to about two orders of magnitude larger than $K_{D}$ (19).

**Fig. 2.**
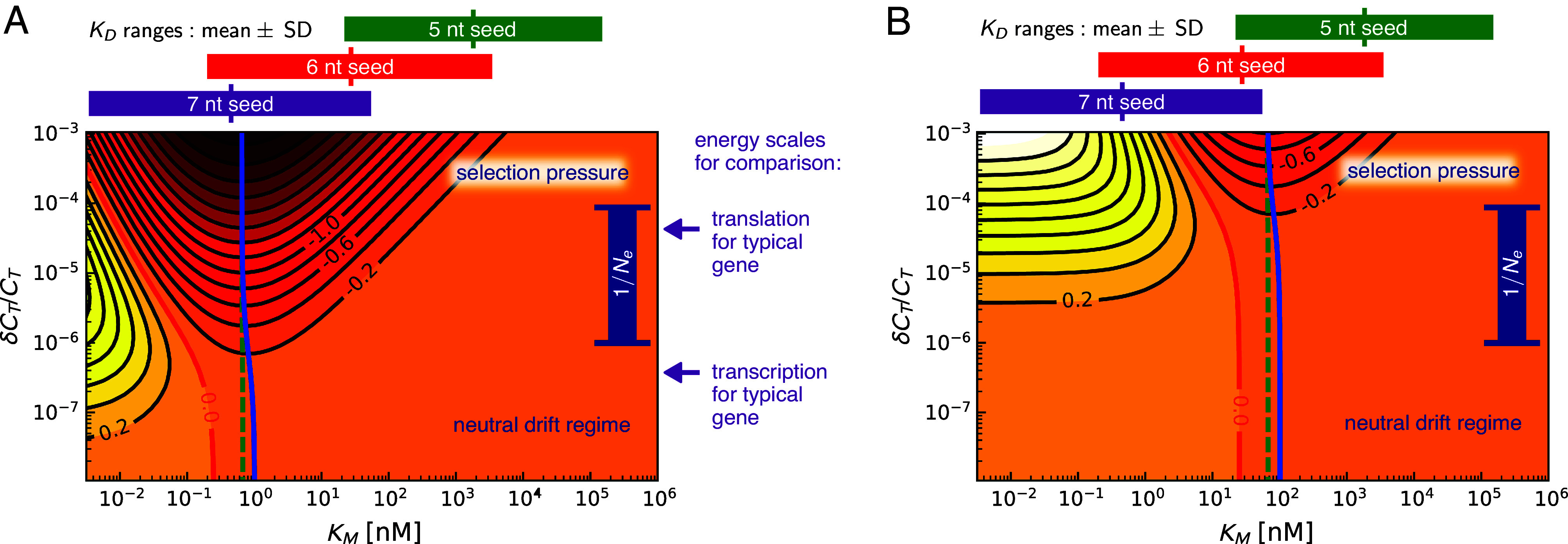
Contour diagrams of noise filter error, $\log_{10}E$, in terms of Michaelis–Menten constant $K_{M}$ and fractional metabolic cost $\delta C_{T}/C_{T}$ for a fixed protein output level $\overset{¯}{p}$ in the (*A*) single-target and (*B*) 100-target cases. The spacing between contour values is 0.2. The minimum contour line for a given $\delta C_{T}/C_{T}$ corresponds to the most energetically efficient noise reduction, and this is achieved by $K_{M}^{*}$ values along the blue curves. Similarly, the green dashed curves show the most efficient $K_{M}^{\mathrm{wk}}$ values predicted by the WK optimal filtering theory. The red line is the boundary of the “dud” region on the left, inside which the miRNA regulation adds noise (E>1) rather than mitigating it. To make a connection to physiological values of $K_{M}$, we plot estimated $K_{D}$ ranges for known miRNA–mRNA interactions above the plots for 5, 6, and 7 nt seed lengths. $K_{D}$ sets a lower bound on $K_{M}$ from Eq. **1**. Altogether, this shows that for a single typical target gene, the most economical noise reduction is likely to occur for 7 nt seeds, while 6 nt seeds become favorable for miRNAs with many targets. To better illustrate the evolutionary relevance, we also plot the dark blue bar showing inverse effective population sizes $N_{e}^{-1}=10^{-6}\;\mathrm{to}\;10^{-4}$ typical for metazoans. As the fitness disadvantage due to metabolic costs becomes significant, $\delta C_{T}/C_{T}≳N_{e}^{-1}$, there is selective pressure on the organism driving it toward the $K_{M}^{*}$ value. For comparison, we also show typical translation and transcription energy scales, based on ref. 18.

In Fig. 2*A*, we show the contour diagram of $\log_{10}E$ as a function of $K_{M}$ and fractional metabolic cost $\delta C_{T}/C_{T}$, assuming a single mRNA target and using the experimentally derived parameters of *SI Appendix*, Table S1. The blue curve denotes the optimal value $K_{M}^{*}$, which achieves the minimum E for a given cost $\delta C_{T}/C_{T}$. The red contour line marks the value $K_{M}^{d}$ where E=1, which corresponds to $F_{R}=F_{0}$. This is the boundary between the noise control region to the right, where E<1 ($F_{R}<F_{0}$) and a “dud” region to the left, where E>1 ($F_{R}>F_{0}$). In the latter region, regulation adds protein noise to the system rather than mitigating it, which can provide an alternative role for some microRNA systems in triggering cell state transitions (20, 21). For a fixed $\delta C_{T}/C_{T}$, as we scan $K_{M}$ from small to large values, we cross from dud to noise control at $K_{M}^{d}$, improve the filter performance until we reach $K_{M}^{*}$, and then get progressively worse filtering for $K_{M}>K_{M}^{*}$. The different behaviors of the system with varying $K_{M}$ reflect the tradeoff due to miRNA–mRNA affinity mentioned earlier in the noise filter discussion. The optimal affinity $K_{M}^{*}$ is a metabolic sweet spot between a regime where miRNA–mRNA interaction is too strong (small $K_{M}$), leading to excessive added noise and the “dud” scenario, and a weak interaction regime (large $K_{M}$) where the miRNA system cannot effectively dampen noise.

In the biologically relevant parameter regime $\gamma_{\mu }$, $\sigma_{\epsilon }$, ϕ≪1, where $\phi \equiv d_{p}/d_{m}$, we can derive analytical approximations for both $K_{M}^{d}$ and $K_{M}^{*}$. For small costs $\delta C_{T}/C_{T}$, we have $K_{M}^{d}\approx \overset{¯}{m}\left(1+\gamma_{\mu }^{-1}\right)$, and with increasing cost the boundary begins to decrease as $K_{M}^{d}\approx \overset{¯}{m}{\left(\left(1-R\right)/\left(R\gamma_{\mu }\right)\right)}^{1/2}$. On the other hand, $K_{M}^{*}$ is remarkably stable as $\delta C_{T}/C_{T}$ is varied. In the large cost limit, it approaches:

[4] $$\begin{array}{c} K_{M}^{*}\approx \overset{¯}{m}\gamma_{\mu }^{-1}\sigma_{\epsilon }^{-1/2}. \end{array}$$

Thus, the optimal affinity depends on two nondimensional biochemical ratios, $\gamma_{\mu }$ and $\sigma_{\epsilon }$, and the mean mRNA target number $\overset{¯}{m}$.

Fig. 2*B* shows what happens when we scale up $\overset{¯}{m}$ by a factor of 100, roughly mimicking the case of 100 similar mRNA targets. As predicted by Eq. **4**, $K_{M}^{*}$ increases by a factor of 100, but the contour levels are pushed up by a similar factor: as expected, it costs more to achieve the same level of noise control when compared to the single-target case. Interestingly, the $K_{M}^{*}$ values in the single and multi-target case (about 0.65 and 65 nM respectively at high $\delta C_{T}/C_{T}$) are comparable to $K_{M}$ measured in a fruit fly RNA interference pathway (19). In the experiments, $K_{M}=1\pm 0.2$ nM was found for a fully complementary interaction with a 7 nt seed, while single nucleotide mismatches in the seed binding (which in principle allow for a larger range of possible targets) boosted $K_{M}$ by up to a factor of 82. While we do not yet have extensive experimental surveys of $K_{M}$ values in miRNA or related siRNA [small interfering RNA] systems, it would be intriguing to check whether $K_{M}$ tends to scale with the target population, as predicted by the optimal theory.

We can make the connection between the number of complementary matches (or seed length) and $K_{M}$ more explicit. In *SI Appendix*, section S.IV, we used the ViennaRNA server (22) to predict the $\Delta G^{0}$ values of $\approx 10^{4}$ human miRNA seed sequences of length 7 nt, resulting in a distribution of $K_{D}$ values which covered around 10 decades on a logarithmic scale. The mean and standard deviation (SD) of $\log_{10}\left(K_{D}/\left[1M\right]\right)=-9.5\pm 2.2$ is shown as a purple bar above the plots in Fig. 2 *A* and *B*. To mimic shorter seeds, we deleted 1 or 2 nucleotides from the sequence, to give the ranges $\log_{10}\left(K_{D}/\left[1M\right]\right)=-7.6\pm 2.0$ (6 nt, red bar) and −5.8±1.9 (5 nt, green bar). As validation, the calculation was able to correctly reproduce measured $K_{D}$ ranges for fully matched 7 nt seeds in fruit fly and mouse siRNA-target complexes. Since $K_{D}$ sets the floor for $K_{M}$, we see from Fig. 2*A* that the optimal $K_{M}^{*}\approx 1$ nM for the single-target case is unlikely to be accessible for 5 nt seeds. It becomes plausible for 6 nt seeds, and even more so for 7 nt seeds. Since $K_{M}$ can be up to two decades larger than $K_{D}$ (19), it is notable that 7 nt seeds densely cover the range of $K_{D}$ values one or two decades smaller than $K_{M}^{*}$. Extrapolating this pattern, longer seeds (≥8 nt) will be less likely than 7 nt to achieve $K_{M}^{*}$. For the 100-target case (Fig. 2*B*), we see that the ideal seed length is shifted to 6 nt as a result of the increase in $K_{M}^{*}$. Seeds of length 6 nt constitute 67% of a dataset of human and mouse miRNA seed sequences, with 7 nt seeds forming another 23% (23). The preponderance of 6 nt seeds is in line with expectations if $K_{M}$ was optimized, particularly since miRNAs will typically have many different targets.

## Noise Reduction Can Approach Optimal Linear Filter Performance.

The filter analogy in Eq. **2** allows us to make an interesting comparison. For a given signal s(t) and n(t), we know that there is a filter function $H_{\mathrm{wk}}\left(t\right)$, the Wiener–Kolmogorov (WK) solution (24–26), which gives the best performance (smallest E) of all possible functions H(t) for this type of linear noise filtering system (*SI Appendix*, section S.II B). We denote the corresponding value of error, $E_{*}^{\text{wk}}$, and it serves as the overall lower bound on E. In our system, E reaches a minimum $E^{*}$ at $K_{M}^{*}$ for a given energetic cost, but is this minimum $E^{*}$ comparable to $E_{*}^{\text{wk}}$? In other words, can miRNA noise regulation approach an optimal WK filter? This type of comparison has recently proven fruitful in a variety of biological contexts (17, 27–33), for example, yielding tight bounds on the fidelity of information transmission in signaling networks.

**Fig. 3.**
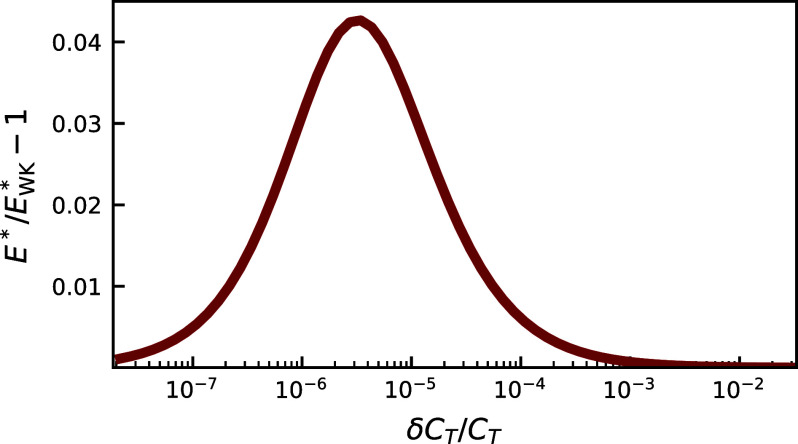
The discrepancy between $E^{*}$ and $E_{\mathrm{wk}}^{*}$, the error of the actual system and the WK optimal system respectively, at the most energetically efficient $K_{M}$ for the single-target case (Fig. 2*A*), at different values of the fractional cost $\delta C_{T}/C_{T}$. $E^{*}/E_{*}^{\text{wk}}-1<0.045$, so the miRNA system exhibits a performance close to WK optimality for this network motif.

The miRNA system does not exactly realize true WK optimality, because the optimal filter function $H_{\mathrm{wk}}\left(t\right)$ cannot be precisely implemented by the miRNA regulation network. However, the affinity $K_{M}^{\text{wk}}$ predicted to be optimal by the WK theory for a given $\delta C_{T}/C_{T}$ (green dashed curve in Fig. 2 *A* and *B*) is extremely close to $K_{M}^{*}$ (blue curve). Fig. 3 shows the difference between the respective errors $E^{*}$ and $E_{*}^{\text{wk}}$ along these curves, as a function of $\delta C_{T}/C_{T}$. While $E^{*}/E_{*}^{\text{wk}}-1>0$, as expected, the difference is always smaller than 0.045, peaking at moderate cost values. As the cost $\delta C_{T}/C_{T}$ increases, $E^{*}$ converges to $E_{*}^{\text{wk}}$, and $K_{M}^{*}$ similarly converges to $K_{M}^{\text{wk}}$. Notably, despite its constraints, the miRNA system can get quite close to WK optimal performance.

## Evolutionary Pressure on miRNA Noise Regulation.

The previous two sections have argued that optimality in noise reduction (in the broader WK sense) is in principle approachable, and 6 to 7 nt seeds put miRNA systems within reach of achieving it. The final question we would like to consider is whether there would be any pressure from natural selection actually driving miRNA regulation toward optimality. From a population genetics perspective, let us say that the fitness of an organism with a particular miRNA regulatory network is $f_{R}$, while the same organism missing the network has fitness $f_{0}$. The selection coefficient $s=f_{R}/f_{0}-1$, quantifying the relative fitness, can be decomposed into two contributions, $s=s_{a}+s_{c}$ (18). Here, $s_{a}$ is the adaptive advantage due to the regulation, for example resulting from protein noise reduction. The remaining part, $s_{c}<0$, comes from the added metabolic cost of implementing the regulation. In order for s to be overall positive (and hence $f_{R}>f_{0}$), we would need an $s_{a}>0$ that is larger in magnitude than the cost, $s_{a}>\left|s_{c}\right|$.

The evolution of miRNA systems, however, poses a conundrum: a newly arisen miRNA, before any selective fine-tuning of its seed sequence, could potentially target hundreds of mRNA in a random manner, making $s_{a}<0$ due to deleterious effects on existing genetic networks. So how would advantageous miRNA regulation eventually emerge? The solution to this problem, as argued by Chen and Rajewsky (2), is for new miRNA systems to be expressed at very low levels, such that $s_{a}$, even if negative, has a negligible magnitude. In this regime, however, $s\approx s_{c}<0$, which is still an overall fitness disadvantage. Would such a deleterious variant survive in a population long enough for further mutations to confer a significant positive $s_{a}$? The answer depends on the magnitude of $|s_{c}|$ relative to a threshold known as the “drift barrier” (34). This threshold is set by $N_{e}^{-1}$, where $N_{e}$ is a measure of genetic diversity known as the effective population size (35). $N_{e}$ is the size of an idealized population that shows the same changes in genetic diversity per generation (due to genetic drift, or random sampling of variants) as the actual population. $N_{e}$ is generally smaller than the real population size and among more complex eukaryotes (like vertebrates) can be as small as $10^{4}$ to $10^{6}$ (18, 36). If $|s_{c}|\ll N_{e}^{-1}$, selection against the variant is weak, even when $s_{c}<0$, so it can survive in a population via genetic drift as an effectively neutral mutant and even eventually take over with a fixation probability roughly given by its initial fraction in the population (37). On the other hand, if $|s_{c}|\gg N_{e}^{-1}$, then the costs are sufficiently high that selection would efficiently weed out the variant from the population, unless there were compensating advantages, $s_{a}≳\left|s_{c}\right|$.

A recent derivation based on a general bioenergetic growth model for organisms allows us to make the above discussion more quantitative: it showed that $s_{c}\approx -\ln\left(R_{b}\right)\delta C_{T}/C_{T}$, where $R_{b}$ is the mean number of offspring per individual (38). Assuming that $\ln(R_{b})$ does not change the order of magnitude, we can thus use the fractional cost $\delta C_{T}/C_{T}$ as a proxy for $|s_{c}|$, and compare it to $N_{e}^{-1}$. The blue range bars on the right in Fig. 2 *A* and *B* show possible $N_{e}^{-1}$ values $10^{-6}\;\mathrm{to}\;10^{-4}$ for higher-order eukaryotes, separating a selection pressure regime at large $\delta C_{T}/C_{T}$ from a neutral drift regime at low $\delta C_{T}/C_{T}$. For comparison, we also show the cost scales for transcription and translation of a typical gene (18), which indicate that transcription is not generally not under selective pressure in these organisms, while translation may be.

At the lowest expression levels, a newly evolved miRNA system could survive in the neutral drift regime, even with a non-optimal $K_{M}$. There is limited noise reduction achievable in this regime, since the contours indicating E significantly smaller than 1 require larger $\delta C_{T}/C_{T}$, particularly for the multi-target case (Fig. 2*B*). Thus, the initial evolution could be imagined as a random walk near the bottom edge of the diagram. Mutations that led to greater expression of an miRNA, moving up on the cost scale, would hit against the drift barrier, and would more likely survive if they came with compensating fitness advantages. In a context where noise control was beneficial, this would mean being funneled up the region where $K_{M}$ is close to $K_{M}^{*}$, the path that confers the largest noise reduction as expression levels rise. Once the costs are above the drift barrier, there would be significant selective pressure to optimize $K_{M}$. In this high-cost regime, there is a direct tradeoff between extra metabolic expenditure and noise filtering, with $\Delta M_{\nu }∼M_{\nu }/E$, and hence $s_{c}∼-M_{\nu }/E$. Our theory predicts that any compensating fitness advantage $s_{a}>0$ would have to grow like $E^{-1}$ or faster as E decreases, in order for the regulation to be viable in the long term.

## Inferring Closeness to Optimality in Experimental Systems.

Given the achievability of optimal affinities $K_{M}^{*}$ for noise filtering (based on seed lengths), and the evolutionary pressures that could drive a system toward this optimality, is there any corroborating experimental evidence? While we do not have simultaneous measurements yet of $K_{M}$ and noise regulation in specific systems, there is a way of approximately inferring the ratio $K_{M}/K_{M}^{*}$ from fitting our model to existing data on miRNA noise suppression. The two experiments we focus on are assays that take the 3’UTR regions of endogenous mRNAs and combine them with fluorescent reporters like *mCherry*—in one case, the 3’UTR was from the *Lats2* gene in mouse embryonic stem cells (5) and in the other from the *sens* gene in *Drosophila* wing disc cells (39). These 3’UTR regions have binding sites for endogenous miRNA, and the assay demonstrates that miRNA interaction suppresses protein noise for these genes, by quantifying reporter protein fluctuations in the wild type compared to a mutant system where the binding sites were altered, inhibiting miRNA regulation. In both cases the noise suppression has potential functional roles—Lats2 is involved in regulating the cell cycle, apoptosis, and differentiation (40, 41), and excess noise in Sens protein levels leads to disordered sensory patterning in wing disc cells (39).

**Fig. 4.**
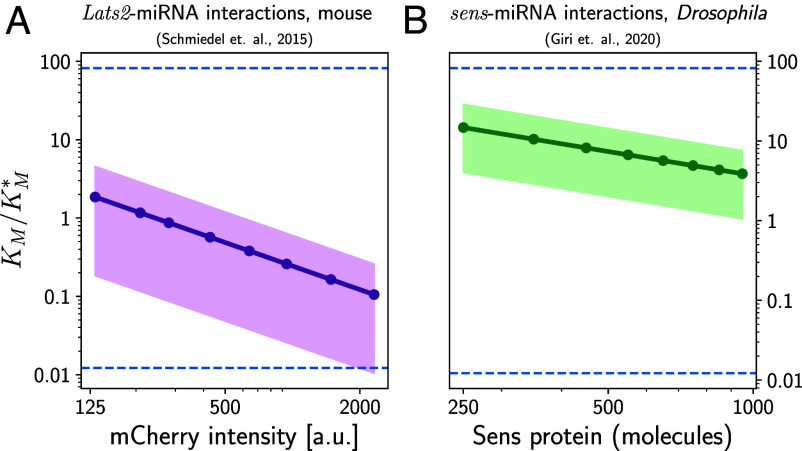
Analysis of optimality for miRNA–mRNA affinity in data from two experimental systems. We show the inferred ratio of actual to optimal Michaelis–Menten constants $K_{M}/K_{M}^{*}$, as a function of protein expression level in individual cells, for miRNA regulation of (*A*) the *Lats2* gene in mouse embryonic stem cells (5); (*B*) the *sens* gene in *Drosophila* wing disc cells (39). The points connected by a line are theoretical best-fits using typical parameter values from *SI Appendix*, Table S1, while the colored regions represent uncertainties due to varying the system parameters $\gamma_{\mu }$ and $\sigma_{\mu }$ over biologically plausible ranges. The horizontal axis reflects different quantification of protein expression in the two experiments, either in fluorescent reporter intensity (*Lats2*) or copy number (*Sens*). The dashed lines represent the maximum positive or negative fold-change in $K_{M}$ observed experimentally from single nucleotide differences in seed matching in ref. 19.

Thus, there is reason to suspect that there could have been selective pressure on the mRNA-miRNA affinity for these genes. To investigate this hypothesis, we took the available experimental data from the two studies and extracted the error E and regulation strength R as a function of protein expression, since there was a distribution of values for $\overset{¯}{p}$ (or reporter intensity) $\propto \overset{¯}{m}$ among the population of cells in each system. (The full details of the data analysis can be found in *SI Appendix*, section S.V). For a given cell, we know that $K_{M}^{*}$ is proportional to the mRNA concentration $\overset{¯}{m}$ from Eq. **4** and thus should be higher in high-expression versus low-expression cells. On the other hand, the types of miRNA and binding sites are the same between cells, so the actual affinity $K_{M}$ should also be the same in each cell. As before, we use a simple version of the multi-miRNA, multi-target theory assuming similar biochemical parameters for each miRNA–mRNA interaction, so we can interpret $K_{M}$ to be an average affinity across the ensemble of miRNAs interacting with the targets. To summarize, $K_{M}$ is fixed but $K_{M}^{*}$ varies between cells, and we can ask whether the ratio $K_{M}/K_{M}^{*}$ is close to 1 (optimality) over the cell population. Fig. 4 shows the results for this ratio as a function of protein expression in the *Lats2* and *sens* systems, derived from fitting our model to the data. The points connected via lines correspond to fits using the typical parameter values in *SI Appendix*, Table S1, while the surrounding colored regions represent uncertainties due to imperfect knowledge of the parameters $\gamma_{\mu }$ and $\sigma_{\epsilon }$ (*SI Appendix*, section S.V). Despite this uncertainty, the inferred $K_{M}/K_{M}^{*}$ ratio is roughly within an order of magnitude of 1, and for the *Lats2* system even crosses 1. The horizontal dashed lines above and below 1 indicate a factor of 82 in either direction, representing the maximum magnitude of fold change in $K_{M}$ observed from a single nucleotide difference in seed matching in another experiment (19). This range emphasizes the relative narrowness of the observed $K_{M}/K_{M}^{*}$ ratio, with the affinity fine-tuned to within a nucleotide of optimality. Moreover, this is true across the whole span of protein expression observed in the experiments, which is also physiologically relevant for these cells. So even though low-expression cells and high-expression cells have different optimal parameters (and error bounds $E^{*}$), they all are fairly close to their respective optima. While there is still work to be done in narrowing down parameter uncertainties and extending the analysis to other systems in future studies, this initial analysis is consistent with evolutionary pressures shaping miRNA–mRNA affinity in systems where noise reduction is important.

## Discussion and Conclusions

Putting all the elements of our theory together, we show that if noise control confers a fitness advantage for a particular miRNA system, there is selective pressure driving it toward an optimal miRNA–mRNA affinity, as described by the Michaelis–Menten constant $K_{M}$. Remarkably, the optimal value $K_{M}^{*}$ in Eq. **4** can be achieved by a fairly narrow range of seed lengths (6 to 7 nt), which happen to make up the vast majority of miRNA seeds. While we argue the plausibility of this key result based on realistic ranges of biological parameters, Eq. **4** opens the way for future experimental validation in specific, fully characterized systems. Such validation should be practical, since the equation only involves a small number of biochemical parameters. The noise reduction at optimality approaches the performance of a Wiener–Kolmogorov filter, the best possible linear noise filter. If true, such optimization would be a striking example of metabolic costs directly shaping the course of evolution for a biochemical network in eukaryotes. This is unusual in itself because eukaryotes are generally less likely to prioritize energy efficiency relative to prokaryotes, which have higher effective population sizes and thus lower drift barriers (18). Beyond the implications for miRNA evolution, the theory could also find applications in the design of synthetic circuits with 3’UTR engineering and artificial miRNAs (42–45).

While the simple theory of miRNA–mRNA interaction used here is sufficient to describe certain experiments (5), there are a variety of model assumptions that can be relaxed in future investigations, to test the conclusions more broadly. For example, one aspect was ignored in the current model: many miRNAs can bind to multiple sites on a single target, with potentially different affinities, as well as exhibit varied affinities more generally across multiple mRNA targets. While we do not expect this heterogeneity to qualitatively change the overall results, it will be interesting to see how it shifts the relations between affinity, metabolic costs, and noise reduction. Multiple binding sites/targets can also lead to nonlinear phenomena like ultrasensitivity and bistability in miRNA–mRNA systems (46), which in turn could give noise reduction additional functional implications, like inhibiting stochastic switching between different cellular states. More complex models can also consider the role of miRNA in mitigating the effects of (possibly nonstationary) environmental fluctuations, along with the noise due to cellular processes (8, 47).

Though our work focuses on noise control via miRNA regulation, it is also important to keep in mind that noise control can be implemented via other mechanisms, and that miRNA themselves have other functional roles. Protein noise can be reduced (maintaining the same expression level) by increasing transcription and either decreasing translation rates or enhancing mRNA degradation (48, 49), and this would avoid noise added due to miRNA interactions (50). Degradation rates could for example be fine-tuned by the length of mRNA poly(A) tails (51). However, this mechanism is nonspecific, while miRNAs allow for selective noise control via seed recognition. Though our results suggest a formative role for noise reduction in shaping miRNA–mRNA affinity, the filtering capacity likely co-evolved with other miRNA functions such gene silencing and cross talk (52). Perhaps in some cases, noise regulation is simply a complementary benefit of a far more complex utility scheme.

Take for instance the scenario where there is selective pressure for gene silencing via miRNA, in other words suppressing the protein level $\overset{¯}{p}$ for a particular gene. Naively, one might expect this pressure to always favor smaller values of $K_{M}$, since higher affinities can provide the same amount of suppression using fewer miRNA (and hence at smaller metabolic cost). But as we saw in our theoretical analysis (i.e., the “dud” regime on the left in the panels of Fig. 2), smaller $K_{M}$ also introduces extra noise into the system. If this noise becomes sufficiently high that it has deleterious effects, like unwanted stochastic transitions between cellular states, then there will be a countervailing pressure to increase $K_{M}$. The balance between these two effects might result in a metabolic sweet spot for affinity, analogous to the one we described in our model, though the posited functional role of the miRNA system was different.

Viral miRNAs present another example of alternative functional roles. These miRNAs exploit the host metabolism and are likely useful for nonnoise-related tasks like evading the host immune response (53). Notably, though viruses lack their own metabolic machinery, there can still be selective pressures on viral miRNA expression as part of the overall energetic costs associated with viral copying (54). Ultimately, a deeper understanding of miRNA evolution will require larger-scale models of its full regulatory context, coupled with in vivo experiments to explore the tangled effects of function, metabolic costs, and fitness.

## Materials and Methods

*SI Appendix* contains full details of the theoretical derivations and data analysis techniques used in our work. *SI Appendix*, section S.I describes the biochemical reaction network for miRNA regulation of mRNA and its formulation in terms of chemical Langevin equations. The key results are analytical expressions for the protein noise as a function of system parameters and an estimate of the bioenergetic costs associated with miRNA regulation. *SI Appendix*, section S.II shows how the dynamics of the miRNA–mRNA system can be interpreted as a noise filter, whose optimality is described by Wiener–Kolmogorov theory. *SI Appendix*, section S.III provides details of how the contour diagrams of Fig. 2 were calculated, while *SI Appendix*, section S.IV describes the use of the ViennaRNA server (22) to estimate the ranges of dissociation constant $K_{D}$ values for different seed lengths included in that figure. Finally, *SI Appendix*, section S.V covers the data analysis techniques used to extract the ratio of actual to optimal Michaelis–Menten constants $K_{M}/K_{M}^{*}$ from experimental measurements, the results of which are shown in Fig. 4.

## Supplementary Material

Appendix 01 (PDF)

## Data Availability

Code related to the calculations, routines for plotting the figures, and all the associated datasets are available at Github ( 55).
