# Supplementary material for: Bioenergetic costs and the evolution of noise regulation by microRNAs
Source: Proc Natl Acad Sci U S A. 2024 Feb 22;121(9):e2308796121. doi: 10.1073/pnas.2308796121 (PMC10907262; doi:10.1073/pnas.2308796121)
Supplement: Supplementary file 1 — Appendix 01 (PDF) [file pnas.2308796121.sapp.pdf]

Supporting Information for

Bioenergetic costs and the evolution of noise regulation by microRNAs

Efe Ilker, Michael Hinczewski

E-mail: ilker@pks.mpg.de, michael.hinczewski@case.edu

This PDF file includes:

Figs. S1 to S4
Table S1
SI References

Contents

S.I Analysis of microRNA-mediated noise regulation 2
A Chemical Langevin formalism of microRNA-mediated gene expression . . . 2
A.1 General birth-death processes . . . 2
A.2 Protein production . . . 2
A.3 Steady-state properties, regulation strength, bound fraction of miRNAs: . . . 3
B Protein expression noise . . . 4
C Bioenergetic costs of miRNA regulation . . . 4

S.II The noise-filter formulation of the microRNA regulated system 5
A Realizing miRNA regulation as a noise filtering mechanism . . . 5
B Wiener-Kolmogorov optimality of protein output noise level . . . 5

S.III Calculation of phase diagrams 6
A Contour plots, fitness costs, and Michaelis-Menten constant . . . 6
B Minimal noise for the same metabolic cost . . . 7

S.IV Estimating dissociation constants K_D for seed sequences of varying length 7

S.V Analysis of experimental microRNA systems 9

S.I. Analysis of microRNA-mediated noise regulation

A. Chemical Langevin formalism of microRNA-mediated gene expression.

A.1. General birth-death processes. We start with a general description of chemical Langevin (CL) dynamics, which is the basis for our theoretical model. A biochemical reaction network consists of N different types of biomolecules with time-dependent populations $n_j(t)$, $j = 1, \ldots, N$. The rate of change of a given population, $dn_j(t)/dt$, can be expressed in terms of a number of possible stochastic reactions, which we will label with an index $\rho = 1, \ldots, r$, where r is the total number of reactions. The CL dynamics of the birth-death process for biomolecule j can be written as (1):

$$\frac{dn_j(t)}{dt} = \sum_{\rho=1}^{r} \Delta_{\rho j}\pi_\rho(t) + \sum_{\rho=1}^{r} \Delta_{\rho j}\sqrt{\pi_\rho(t)}\eta_\rho(t) \qquad \text{[S1]}$$

where $\Delta_{\rho j}$ are the stoichiometric coefficients representing the change in the population of j in reaction ρ, $\pi_\rho(t)$ are the rates of reactions and $\eta_\alpha(t)$ are independent Gaussian noise terms at time t which satisfy $\langle \eta_\rho(t)\eta_\beta(t')\rangle = \delta_{\rho\beta}\delta(t-t')$ where $\delta_{\rho\beta}$ and $\delta(t-t')$ are Kronecker delta and Dirac delta functions, respectively. As the reactions for the production and degradation of j are uncorrelated, we can add up the uncorrelated noise terms, and obtain:

$$\frac{dn_j(t)}{dt} = \sum_{\rho=1}^{r} \Delta_{\rho j}\pi_\rho(t) + \sqrt{\alpha_j(t)}\eta_j(t) \qquad \text{[S2]}$$

where $\alpha_j(t) = \sum_{\rho=1}^{r} \Delta_{\rho j}^2 \pi_\rho(t)$. Note that though the η_j's are uncorrelated at different times, there can be non-zero correlations between the dynamics of different populations at time t if they share the same reactions.

A.2. Protein production. To apply the CL framework to microRNA regulation of gene expression, we focus on the following biomolecular species and reactions, following the model of Ref. (2):

$$\varnothing \underset{d_m}{\overset{\nu_R}{\rightleftarrows}} m$$

$$m \xrightarrow{k_p} m + p$$

$$p \xrightarrow{d_p} \varnothing$$

$$\varnothing \underset{d_\mu}{\overset{\nu_\mu}{\rightleftarrows}} \mu$$

$$m + \mu \underset{k_\mathrm{off}}{\overset{k_\mathrm{on}}{\rightleftarrows}} m_b$$

$$m_b \xrightarrow{d_{m\mu}} \mu$$

$$m_b \xrightarrow{d_\mu} m$$

Here m, p, μ, m_b represent respectively mRNA, protein, miRNA and the miRNA-mRNA complex. These reactions include birth-death processes of individual species as well as the formation and degradation of complexes. When the miRNA-mRNA complex m_b degrades, the miRNAs are recycled. For convenience, we assume a similar property when miRNAs spontaneously degrade. This model has been shown to agree well with experimental systems of miRNA-mediated gene expression and noise reduction (2, 3).

The reactions above give the following mass-action kinetics for the concentrations of the four species (denoted by brackets):

$$\frac{d[\mu]}{dt} = \nu_\mu - d_\mu[\mu] - k_{\text{on}}[m][\mu] + (k_{\text{off}} + d_{m\mu})[m_b] \quad \text{[S3a]}$$

$$\frac{d[m]}{dt} = \nu_R - d_m[m] - k_{on}[m][\mu] + (k_{\text{off}} + d_\mu)[m_b] \quad \text{[S3b]}$$

$$\frac{d[m_b]}{dt} = k_{\text{on}}[m][\mu] - (k_{\text{off}} + d_{m\mu} + d_\mu)[m_b] \quad \text{[S3c]}$$

$$\frac{d[p]}{dt} = k_p[m] - d_p[p]. \quad \text{[S3d]}$$

The total miRNA concentration is a sum of free and bound miRNAs, i.e., $[\mu_{tot}] = [\mu] + [m_b]$. Assuming the binding/unbinding rate of the mRNA-microRNA complex is much faster than other dynamics, we obtain a quasi-steady-state approximation leading to $[m_b] = \frac{[m][\mu_{tot}]}{K_{\text{M}}+[m]}$, with Michaelis-Menten constant $K_{\text{M}} = K_{\text{D}} + k_{\text{cat}}/k_{\text{on}}$ expressed in terms of the dissociation constant $K_{\text{D}} = k_{\text{off}}/k_{\text{on}}$ and an effective catalytic rate constant $k_{\text{cat}} = d_{m\mu} + d_\mu$ for the miRNA-catalyzed degradation reaction. With these simplifications, we obtain

$$\frac{d[\mu_{tot}]}{dt} = \nu_\mu - d_\mu[\mu_{tot}] \quad \text{[S4a]}$$

$$\frac{d[m]}{dt} = \nu_R - d_m[m] - \frac{d_{m\mu}}{K_{\text{M}} + [m]}[\mu_{tot}][m] \quad \text{[S4b]}$$

$$\frac{d[p]}{dt} = k_p[m] - d_p[p]. \quad \text{[S4c]}$$

Note that the description in terms of chemical concentrations ignores the fact that the crowded cell interior is not an ideal solution, and that in principle the model can be made more accurate by replacing concentrations with chemical activities in all the reaction rate terms. However, the activity coefficients depend on the concentrations of all chemical species in the cytoplasm (including species not explicitly present in the model), which makes mathematical analysis intractable. The simplest approximation is to assume constant activity coefficients, based on the steady-state values of all the concentrations. In this scenario the activity coefficients can be absorbed into effective rate constants, and the structure of the kinetic equations above stays the same.

Converting concentrations to copy numbers (denoted without brackets) by multiplying with the cell volume V, and using Eqs. [S1]-[S2], we can now introduce the corresponding CL equations for the model:

$$\frac{d\mu_{tot}(t)}{dt} = \nu_\mu - d_\mu \mu_{tot}(t) + \sqrt{\alpha_\mu}\eta_\mu(t) \quad \text{[S5a]}$$

$$\frac{dm(t)}{dt} = \nu_R - d_m m(t) - \frac{d_{m\mu}}{K_{\text{M}} + m(t)}\mu_{tot}(t)m(t) + \sqrt{\alpha_m}\eta_m(t) + \sqrt{\alpha_b}\eta_b(t) \quad \text{[S5b]}$$

$$\frac{dp(t)}{dt} = k_p m(t) - d_p p(t) + \sqrt{\alpha_p}\eta_p(t), \quad \text{[S5c]}$$

where $\langle \eta_i(t)\eta_j(t')\rangle = \delta_{ij}\delta(t-t')$. We will provide a detailed derivation of the noise amplitudes $\alpha_j(t)$ in Section B.

A.3. Steady-state properties, regulation strength, bound fraction of miRNAs:. At the steady-state, the mean populations for our dynamical system are $\bar{p} = \frac{k_p}{d_p}\bar{m}$, $\bar{\mu}_{\text{tot}} = \nu_\mu/d_\mu$, $\bar{m} = \frac{\nu_R}{d_m}(1-R)$, where the regulation strength R is defined as:

$$R = \frac{\theta_\mu \bar{\mu}_{\text{tot}}}{\theta_\mu \bar{\mu}_{\text{tot}} + \gamma_m \bar{m}}. \quad \text{[S6]}$$

Here $\gamma_m \equiv d_m/d_{m\mu}$ and θ_μ is the bound fraction of miRNAs,

$$\theta_\mu \equiv \frac{\bar{m}_b}{\bar{\mu}_{\text{tot}}} = \frac{\bar{m}}{K_{\text{M}} + \bar{m}}. \quad \text{[S7]}$$

In order to compare protein noise at different levels of miRNA regulation (including the no regulation case), we fix the mean protein population by assuming the mRNA production rate ν_R changes with R to compensate for the effects of regulation,

$$\nu_R = \frac{\nu_0}{1-R} = \frac{d_m \bar{m}}{1-R} \quad \text{[S8]}$$

where $\nu_0 = \nu_R(R=0)$.

B. Protein expression noise. The fluctuating molecular populations are defined in the main text as $x(t) = \bar{x} + \delta x(t)$ where $\delta x(t)$ is the variation from the mean level $\bar{x}$ and the species of interest are $x(t) \equiv \{\mu_{tot}(t), m(t), p(t)\}$. Since we focus on the stationary-state fluctuations, we may treat the noise amplitudes α_j as time-independent. We can write these in terms of the equilibrium populations,

$$\alpha_\mu = 2d_\mu \bar{\mu}_{\text{tot}}, \;\; \alpha_m = 2d_m \bar{m}, \;\; \alpha_b = \alpha_m R/(1-R), \;\; \alpha_p = 2d_p \bar{p}, \qquad \text{[S9]}$$

where $\bar{\mu}_{\text{tot}}$, $\bar{m}$, and $\bar{p}$ are mean steady-state population levels for miRNA, mRNA and protein respectively. Using the linear-noise approximation in Eq. [S5] and taking the Fourier transform leads to

$$\delta\mu(\omega) = \frac{\sqrt{\alpha_\mu}\eta_\mu(\omega)}{d_\mu - i\omega} \qquad \text{[S10a]}$$

$$\delta m_R(\omega) = \frac{\sqrt{\alpha_m}\eta_m(\omega) + \sqrt{\alpha_m R/(1-R)}\eta_m(\omega) - d_{m\mu}\theta_\mu \delta\mu(\omega)}{c_m - i\omega} \qquad \text{[S10b]}$$

$$\delta p_R(\omega) = \frac{k_p \delta m_R(\omega)}{d_p - i\omega} + \frac{\sqrt{\alpha_p}\eta_p(\omega)}{d_p - i\omega}, \qquad \text{[S10c]}$$

with $c_m = d_m(1 - R\theta_\mu)/(1-R)$. In Fourier space, the noise correlations satisfy $\langle \eta_i(\omega)\eta_j(\omega')\rangle = 2\pi\delta_{ij}\delta(\omega + \omega')$. The limit $R \to 0$ corresponds to an unregulated system, with $c_m \to d_m$ and $d_{m\mu}\theta_\mu\delta\mu(\omega) \to 0$ in Eq. [S10b].

The main measure that we use to assess noise levels in protein populations is the Fano factor $F \equiv \sigma_p^2/\bar{p}$ where the variance $\sigma_p^2 \equiv \langle (\delta p(t))^2 \rangle = \langle (\delta p(0))^2 \rangle = \int_{-\infty}^{\infty} \frac{d\omega}{2\pi} \langle (\delta p_R(\omega)\delta p_R(-\omega))\rangle$. Using Eq. [S10c] and the correlation properties of the noise terms $\eta_i(\omega)$ in Fourier space, we can calculate the Fano factors F_0 and F_R for the unregulated and regulated systems respectively:

$$F_0 = 1 + \frac{k_p}{d_m + d_p}, \qquad \text{[S11a]}$$

$$F_R = 1 + \frac{k_p}{(c_m + d_p)(1 - R\theta_\mu)} + \frac{d_{m\mu}\theta_\mu(c_m + d_\mu + d_p)k_p R}{(c_m + d_\mu)(c_m + d_p)(d_\mu + dp)(1 - R\theta_\mu)}. \qquad \text{[S11b]}$$

The third term in F_R is a result of coupling with microRNA pool noise (extrinsic noise). We can quantify the relative protein noise levels in an miRNA-regulated system versus an unregulated one with the quantity

$$E = \frac{F_R - 1}{F_0 - 1}. \qquad \text{[S12]}$$

To simplify our analysis, we define the following nondimensional parameters:

$$\gamma_m \equiv \frac{d_m}{d_{m\mu}}, \;\; \gamma_\mu \equiv \frac{d_\mu}{d_{m\mu}}, \;\; \phi \equiv \frac{d_p}{d_m}. \qquad \text{[S13]}$$

Assuming the degradation rate constant of proteins is slower than that of RNAs, i.e. $\phi \ll 1$, we can derive an approximate expression for E,

$$E \approx \frac{(1-R)(\gamma_\mu + R\theta_\mu)}{\gamma_\mu(1 - R\theta_\mu)^2}. \qquad \text{[S14]}$$

C. Bioenergetic costs of miRNA regulation. To estimate bioenergetic costs, we follow the Lynch & Marinov analysis (4), where the transcriptional cost of a typical gene in eukaryotic cells is given by $C_\nu \equiv c_g + M_\nu t_r$. Here c_g are costs due to synthesis (growth), M_ν is the energy consumption rate for maintenance, and t_r is the lifetime of the cell. For long enough cell-division times, the maintenance term dominates, and hence $C_\nu \approx M_\nu t_r$. For the unregulated system, this amounts to $M_\nu = \nu_0 \epsilon_m$, where ϵ_m is the maintenance energy cost per mRNA (i.e. the turnover cost of recycling its nucleotides after degradation into new mRNA). By contrast, the cost for the miRNA regulated system consists of two contributions: increased transcription of mRNAs in order to maintain $\bar{p}$ and transcription of miRNAs. The additional cost relative to the unregulated case, ΔM_ν, can be expressed as $\Delta M_\nu = (\nu_R - \nu_0)\epsilon_m + \nu_\mu \epsilon_\mu$, where ϵ_μ is the maintenance cost per miRNA. By substituting $\nu_R = \nu_0/(1-R)$, $\nu_\mu = d_\mu \bar{\mu}_{\text{tot}}$, $\nu_0 = d_m \bar{m}$ from the definitions in Section A.3, and inverting Eq. [S6] for $\bar{\mu}_{\text{tot}}$, we obtain Eq. (3) in the main text,

$$\Delta M_\nu = \frac{R}{1-R}\left(1 + \frac{\gamma_\mu \sigma_\epsilon}{\theta_\mu}\right) M_\nu, \qquad \text{[S15]}$$

with $\sigma_\epsilon \equiv \epsilon_\mu/\epsilon_m$.

In order to determine the noise levels for a given cost ΔM_ν, we can invert the above equation for R, and then plug into Eq. [S14]. In the limit $\phi \ll 1$ we get

$$E(\mathcal{M}, \theta_\mu) \approx \frac{(\gamma_\mu\sigma_\epsilon + \theta_\mu)(\mathcal{M}\theta_\mu^2 + \gamma_\mu^2\sigma_\epsilon + \gamma_\mu\theta_\mu(1 + \mathcal{M}))}{\gamma_\mu\left(\gamma_\mu\sigma_\epsilon + \theta_\mu(1 + \mathcal{M}(1 - \theta_\mu))\right)^2}, \qquad \text{[S16]}$$

where $\mathcal{M} = \Delta M_\nu/M_\nu$.

S.II. The noise-filter formulation of the microRNA regulated system

A. Realizing miRNA regulation as a noise filtering mechanism. MicroRNA regulation acts directly on mRNA-related fluctuations in protein output production. We can formulate noise regulation as a filtering process by decomposing the protein fluctuations given in Eq. [S10c] as

$$\delta p_R(\omega) = \underbrace{\frac{k_p \delta m_R(\omega)}{d_p - i\omega}}_{s(\omega)-\tilde{s}(\omega)} + \underbrace{\frac{\sqrt{\alpha_p}\eta_p(\omega)}{d_p - i\omega}}_{\delta p_g(\omega)}, \tag{S17}$$

where $\delta p_g(\omega)$ is the ground noise in protein production (the intrinsic noise independent of mRNA fluctuations), and $s(\omega) = \frac{k_p \delta m_R(\omega)}{d_p - i\omega}|_{R=0}$ is the contribution from mRNA noise in the absence of regulation. The perturbation due to regulation, $-\tilde{s}(\omega)$, can then be interpreted in the noise filter framework as an "estimate" of the signal $s(\omega)$, with maximum reduction of the mRNA-induced noise occuring when the estimate is perfect, $\tilde{s}(\omega) = s(\omega)$. Note that $\tilde{s}(\omega)$ and $s(\omega)$ have non-zero correlations, while $\langle(\delta p_g(\omega) s(-\omega))\rangle = \langle(\delta p_g(\omega)\tilde{s}(-\omega))\rangle = 0$. Hence miRNA regulation can compensate for the mRNA noise, but not the ground noise. The normalized error in estimation is defined in the time domain,

$$E = \frac{\langle (s(t) - \tilde{s}(t))^2 \rangle}{\langle s(t)^2 \rangle} \tag{S18}$$

which is equivalent to Eq. [S12] since $\langle (s(t) - \tilde{s}(t))^2\rangle = (F_R - 1)\bar{p}$ and $\langle s(t)^2 \rangle = (F_0 - 1)\bar{p}$. For a general linear filtering problem we can write the estimated signal as

$$\tilde{s}(\omega) = H(\omega)(s(\omega) + n(\omega)), \tag{S19}$$

where $s(\omega) + n(\omega) \equiv c(\omega)$ is the signal corrupted with noise $n(\omega)$, and $H(\omega)$ is the linear filter function. Eq. (2) of the main text is the inverse Fourier transform of Eq. [S19], showing filtering as a convolution in the time domain,

$$\tilde{s}(t) = \int_{-\infty}^{t} dt' H(t - t')(s(t') + n(t')). \tag{S20}$$

The fact that the upper limit of the integral is t reflects an additional physical constraint on the filter: in the time domain $H(t) = 0$ for all $t < 0$. This enforces causality, namely that the estimate $\tilde{s}(t)$ can only depend on on the past history ($t' < t$) of the noise-corrupted signal $s(t') + n(t')$. In Fourier space, this translates to the requirement that $H(\omega)$ extended to complex ω can have no poles or zeros in the upper half-plane, $\mathrm{Im}\,\omega > 0$.

For our system, each of terms in Eq. [S19] can be identified using Eqs. [S17], [S10b], and [S10a]:

$$H(\omega) \equiv \frac{B - A}{A} \tag{S21}$$

$$s(\omega) \equiv \frac{k_p \sqrt{\alpha_m}\eta_m(\omega)}{DA} \tag{S22}$$

$$n(\omega) \equiv -\frac{k_p}{(B - A)D}\left(\sqrt{\alpha_m R/(1 - R)}\eta_b(\omega) - \frac{d_{m\mu}\theta_\mu}{C}\sqrt{\alpha_\mu}\eta_\mu(\omega)\right) \tag{S23}$$

$$c(\omega) \equiv s(\omega) + n(\omega) \tag{S24}$$

with complex functions

$$A \equiv d_m - i\omega, \quad B \equiv c_m - i\omega, \quad C \equiv d_\mu - i\omega, \quad D \equiv d_p - i\omega. \tag{S25}$$

The noise term $n(\omega)$ captures the additional fluctuations due to miRNA-mRNA interactions and it is uncorrelated with the signal $s(\omega)$.

B. Wiener-Kolmogorov optimality of protein output noise level. Wiener-Kolmogorov (WK) theory provides a framework to calculate the optimal causal filter function $H_{\text{wk}}(\omega)$ that minimizes the error E, and thus represents the fundamental limit on how much linearized regulation can reduce mRNA noise. An overview of the WK formalism can be found in Ref. (5). Here we show how to apply it to the miRNA system.

In order to determine the error function E in Eq. [S18], we need to calculate the variance given in the numerator:

$$\langle (s(t) - \tilde{s}(t))^2 \rangle|_{t=0} = \int_{-\infty}^{\infty} \frac{d\omega}{2\pi}\left(|H(\omega)|^2 P_{nn}(\omega) + |1 - H(\omega)|^2 P_{ss}\right) \tag{S26}$$

where the power spectra (or cross-spectral density) functions are defined as:

$$2\pi P_{xy}(\omega)\delta(\omega + \omega') = \langle x(\omega) y(\omega') \rangle \tag{S27}$$

for x, $y = s$ or n. The inverse Fourier transform of this expression would give the cross correlations in the time domain, $C_{xy}(t) \equiv \langle x(t') y(t' + t)\rangle$. The WK optimal filter minimizing E for a given $s(t)$, i.e., minimizing Eq. [S26], satisfies

$$H_{\text{wk}}(\omega) = \frac{1}{P^+_{cc}(\omega)}\left\{\frac{P_{cs}(\omega)}{P^-_{cc}(\omega)}\right\}_+ . \tag{S28}$$

The above expression requires two types of decomposition into causal (+) and anti-causal (−) parts, where a causal term in Fourier space has no poles or zeros in the upper complex ω half-plane, and an anti-causal term is the complex conjugate of a causal function. The first decomposition is multiplicative, where we write $P_{cc} = P_{cc}^+ P_{cc}^-$, a product of causal and anti-causal terms. The second decomposition is additive, denoted with brackets as $\left\{P_{cs}/P_{cc}^-\right\}_+$. Here $\{G(\omega)\}_+$ for a function $G(\omega)$ can be calculated by doing a partial fraction expansion of $G(\omega)$ can keeping only terms with no poles in the upper half-plane.

The relevant power spectra are obtained by inserting Eqs. [S22], [S23], [S24] into Eq. [S27]:

$$P_{cs} = \frac{{k_p}^2}{|D|^2|A|^2}\alpha_m, \qquad P_{cc} = \frac{P_{cs}}{|C|^2 d_m^2}\mathcal{L}\left(\frac{d_m^2|C|^2}{\mathcal{L}} + |A|^2|C|^2 + d_m^2 b_\mu |A|^2\right) \tag{S29}$$

and $P_{ss} = P_{cs}$, $P_{nn} = P_{cc} - P_{cs}$ with dimensionless parameters $b_\mu = \frac{\alpha_\mu(1-R)}{\alpha_m R}(\theta_\mu/\gamma_m)^2$ and $\mathcal{L} = \frac{1-R}{R(1-\theta_\mu)^2}$. To achieve the causal decompositions required for WK equation (S28), P_{cc} can be rewritten as,

$$P_{cc} = \frac{P_{cs}}{|C|^2 d_m^2}\mathcal{L}(\lambda_1 + i\omega)(\lambda_1 - i\omega)(\lambda_2 + i\omega)(\lambda_2 - i\omega) \tag{S30}$$

where $\pm i\lambda_{1,2}$ are the roots of the numerator and given by

$$\lambda_{1,2} = d_m\left(\frac{\Gamma \pm \sqrt{\Gamma^2 - 4\beta}}{2}\right), \quad \Gamma = b_\mu + \left(\frac{\gamma_\mu}{\gamma_m}\right)^2 + \frac{1+\mathcal{L}}{\mathcal{L}}, \quad \beta = b_\mu + \frac{1+\mathcal{L}}{\mathcal{L}}\left(\frac{\gamma_\mu}{\gamma_m}\right)^2 \tag{S31}$$

Thus, we obtain

$$P_{cc}^+ = \frac{P_{cs}^+}{C d_m}\sqrt{\mathcal{L}}(\lambda_1 - i\omega)(\lambda_2 - i\omega) \tag{S32a}$$

$$\frac{P_{cs}}{P_{cc}^-} = \frac{P_{cs}^+ C^* d_m}{\sqrt{\mathcal{L}}(\lambda_1 + i\omega)(\lambda_2 + i\omega)}. \tag{S32b}$$

where $P_{cs}^+ = \frac{k_p\sqrt{\alpha_m}}{DA}$. Next, we need to determine the additive decomposition of P_{cs}/P_{cc}^-, yielding the casual part

$$\left\{\frac{P_{cs}}{P_{cc}^-}\right\}_+ = \sqrt{\frac{\alpha_m}{\mathcal{L}}}\frac{d_m k_p}{(d_m - d_p)}\left(\frac{d_p + d_\mu}{(d_p+\lambda_1)(d_p+\lambda_2)(d_p - i\omega)} - \frac{d_m + d_\mu}{(d_m+\lambda_1)(d_m+\lambda_2)(d_m - i\omega)}\right). \tag{S33}$$

The optimal WK filter can be calculated from Eq. [S28] by inserting Eqs. [S32a] and [S33], resulting in

$$H_{\rm wk}(\omega) = \frac{d_m^2(d_m s_m - d_p s_p)}{\mathcal{L} d_m d_p (d_m - d_p)}\frac{(d_\mu - i\omega)(\frac{d_m d_p(s_m - s_p)}{d_m s_m - d_p s_p} - i\omega)}{(\lambda_1 - i\omega)(\lambda_2 - i\omega)} \tag{S34a}$$

$$\text{where} \quad s_m = \frac{d_p(d_m + d_\mu)}{(d_m+\lambda_1)(d_m+\lambda_2)}, \tag{S34b}$$

$$s_p = \frac{d_m(d_p + d_\mu)}{(d_p+\lambda_1)(d_p+\lambda_2)}. \tag{S34c}$$

The filter $H_{\rm wk}(\omega)$ contains two characteristic frequencies λ_1, λ_2. This differs from the functional form of $H(\omega)$ in Eq. [S21] which has a single characteristic frequency d_m^{-1}. To evaluate how close the miRNA-mediated noise regulation can approach WK optimality, we numerically compare the errors $E, E_{\rm wk}$ of both cases respectively. We get the error in estimation in WK optimality using Eq. [S26] in Eq. [S18] with $H(\omega) \to H_{\rm wk}(\omega)$,

$$E_{\rm wk} = \frac{F_{\rm wk} - 1}{F_0 - 1} = \frac{d_m^2}{\mathcal{L}(d_m - d_p)^2}\left((d_m + d_p)\left(\frac{s_m^2}{d_p} + \frac{s_p^2}{d_m}\right) - 4 s_m s_p\right). \tag{S35a}$$

Fig. S1 illustrates the discrepancy between $E_{\rm wk}$ from the above equation and E from Eq. [S12].

S.III. Calculation of phase diagrams

A. Contour plots, fitness costs, and Michaelis-Menten constant. In the main text, we plot the contour diagram of $\log_{10} E$ from Eq. [S12], describing noise reduction for miRNA-regulated gene expression at a fixed protein output $\bar{p}$. The gene expression parameters are given in Table S1, while the Michaelis-Menten constant $K_{\rm M}$ and miRNA levels $\mu_{\rm tot}$ are taken as free parameters which control both noise reduction and the metabolic costs of regulation. The regulation costs are calculated using Eq. [S15], and the definitions of energetic costs per mRNA and per miRNA respectively,

$$\epsilon_m = \zeta_P L_{m,\rm pre}, \qquad \epsilon_\mu = \zeta_P L_{\mu,\rm pri} \tag{S36}$$

where ζ_P is the energetic need per nucleotide (which we express in units of phosphate bonds hydrolyzed), and $L_{m,\rm pre}$, $L_{\mu,\rm pri}$ are the numbers of nucleotides of precursor mRNA and primary miRNA. In principle, the costs associated with the miRISC can be larger than ϵ_μ. Thus, our estimations can be seen as a lower bound. Finally, we relate metabolic costs to a fitness disadvantage, $s_c \sim -\Delta M_\nu / M_{\rm tot}$ where $M_{\rm tot}$ is the (mean) total metabolic rate of a cell, as discussed in the main text.

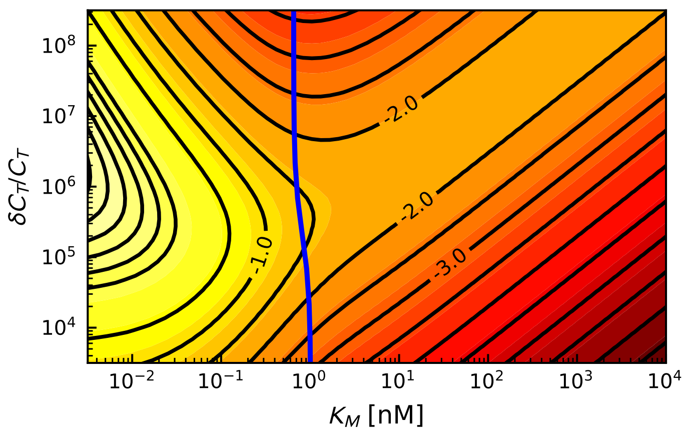

Fig. S1. Contour diagrams of $\log_{10}(E/E_{\mathrm{wk}}-1)$, in terms of Michaelis-Menten constant K_{M} and fractional metabolic cost $\delta C_T/C_T$ for a fixed protein output level $\bar{p}$ in the single target case using the parameters from Table S1. The contour spacing is 0.5, and the blue line follows the most energetically efficient noise reducing K^*_{M} as in Fig. 2 of the main text.

Parameter	Definition	Value	Reference
d_m	mRNA degradation rate constant	0.11 hr^{-1} per mRNA	(6)
d_p	protein degradation rate constant	0.022 hr^{-1} per protein	(6)
d_μ	microRNA degradation rate constant	0.05 hr^{-1} per miRNA	(7, 8)
$d_{m\mu}$	mRNA degradation through miRISC	0.44 hr^{-1} per complex	
K_{M}	Michaelis-Menten const. for target degradation	solved for	
$L_{m,\mathrm{pre}}$	precursor mRNA nucleotide number	20,000	(9)
$L_{\mu,\mathrm{pri}}$	primary miRNA nucleotide number	2,500	(10, 11)
ζ_P	P bonds hydrolyzed per nucleotide recycling	2.17 P hr^{-1}	(4)
M_{tot}	total metabolic rate per cell	3×10^{11} P hr^{-1}	(4)
V	volume of cells	2,000 $\mu\mathrm{m}^3$	(12)
ϕ	d_p/d_m	0.2	
γ_m	$d_m/d_{m\mu}$	0.25	
γ_μ	$d_\mu/d_{m\mu}$	0.114	
$\bar{m}$	mean mRNA copy number	31	(6, 12)
$\bar{p}$	mean protein copy number	200,000	(6, 12)
σ_ϵ	ϵ_μ/ϵ_m	0.125	

Table S1. Gene expression parameters (metazoan) used in calculating the phase diagrams. Typical (or estimated) values from the corresponding references are taken such as median values over a distribution of genes. When an extensive distribution is not present, a reasonable estimation is made compatible with the references. We have not found a value for $d_{m\mu}$ in the literature, however, target mRNAs degrade faster in the complex, and hence we assumed the ratio to be within an order of magnitude. In order to convert the copy number units to molar units of mRNA numbers and K_{m}, we divide these values by the cell volume V and Avagadro constant.

B. Minimal noise for the same metabolic cost. The contour plots indicate a minimum E for a given metabolic cost $\sim \Delta M_\nu$ at a precise K_{M} value. This value remains in a narrow range as a function of metabolic costs as shown in the Fig. 2 of the main text. Taking the limit $\phi \ll 1$, we can estimate this value by minimizing Eq. [S16] with respect to $\theta_\mu(K_{\mathrm{M}})$. Among multiple solutions, there is a single physical one, i.e., $0 \le \theta_\mu(K^*_{\mathrm{M}}) \le 1$, from which we can infer K^*_{M} using Eq. [S7]. For high $\mathcal{M}$ and $\gamma_\mu \ll 1$, this yields the Eq. 4 of the main text,

$$K^*_{\mathrm{M}}(\Delta M_\nu, \bar{p}) \approx \bar{m}\gamma_\mu^{-1}\sigma_\epsilon^{-1/2}. \qquad \text{[S37]}$$

This approximation holds well for the parameters in Table S1, with only 0.6% discrepancy at high $\mathcal{M}$. For $\sigma_\epsilon \ll 1$ we observe that $E(K^*_{\mathrm{M}}) \sim \mathcal{M}^{-1}$, which relates noise reduction to metabolic and hence to the fitness costs, i.e., $s_c \propto -M_\nu/E(K^*_{\mathrm{M}})$.

S.IV. Estimating dissociation constants K_{D} for seed sequences of varying length

To estimate a range of possible K_{D} values for miRNAs with different seed sequences, we started with a collection of 9990 human miRNA seed sequences of length 7 nucleotides (nt) taken from the TargetScanHuman database, ver. 7.1 (14). For each sequence, we used the RNAcofold algorithm from the ViennaRNA package (ver. 2.3) to calculate the free energy ΔG^0 at $T = 298°\mathrm{K}$ for binding to a complementary target sequence (13). To mimic the effects of unpaired nucleotides flanking the seed-target complex, we added two random unpaired nucleotides at both the beginning and end of the seed/target to make the

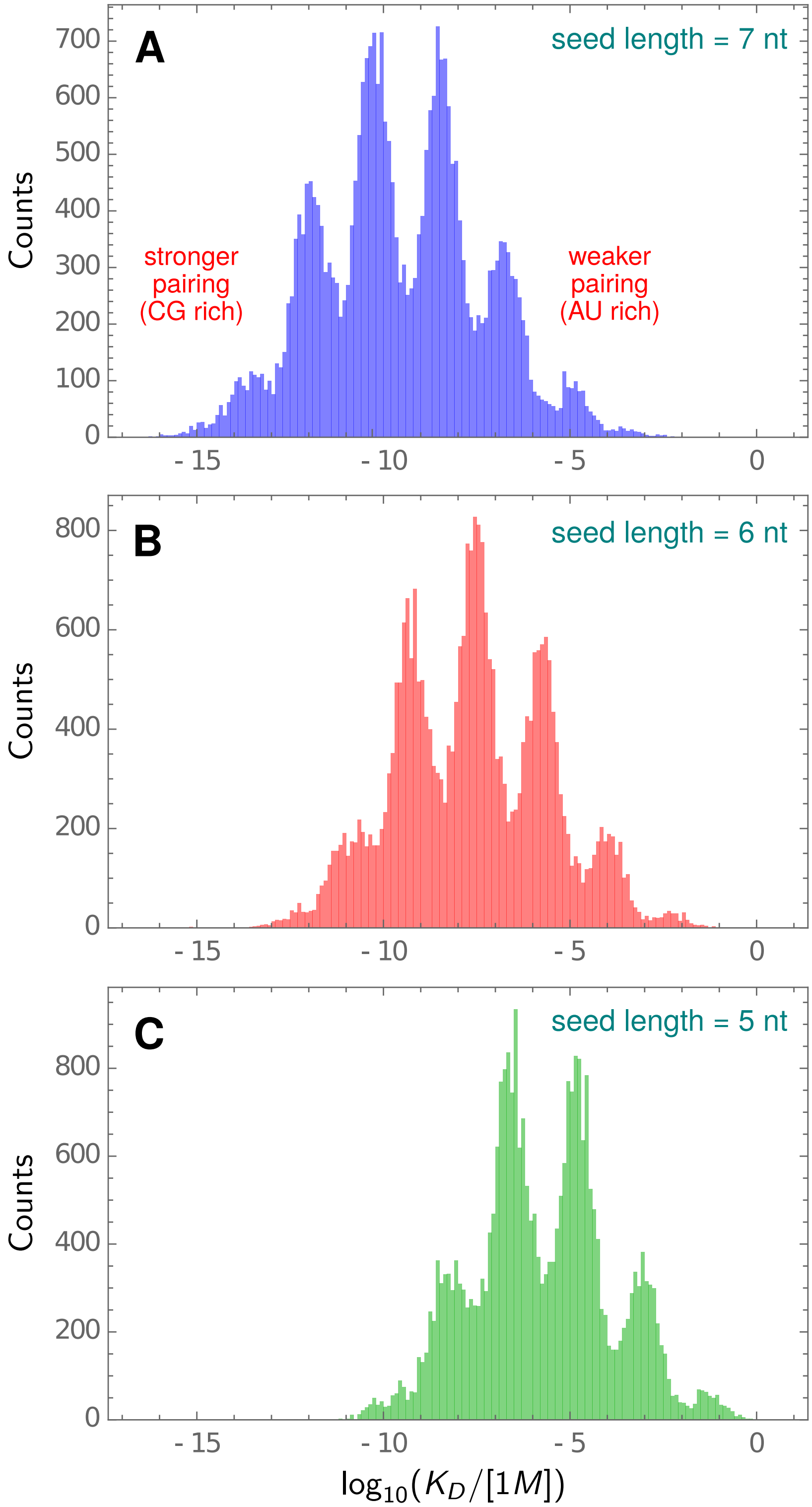

Fig. S2. Distribution of K_{D} values estimated for a different miRNA seed sequences with length: A) 7 nt; B) 6 nt; C) 5 nt. The calculation used the RNAcofold algorithm of the ViennaRNA package (13).

total length 11 nt. The algorithm was run with the default dangling end energy option. Once ΔG^0 is known, $K_{\rm D}$ can be found using the relation $\Delta G^0 = k_B T \ln(K_{\rm D}/[1M])$.

To validate the predictions of the algorithm, we checked it on the seed sequence GAGGUAG, for which experimental $K_{\rm D}$ values are available for fruit fly and mouse siRNA-target complexes (15). Depending on the dangling ends, the algorithm predicted a range of $K_{\rm D}$ with mean 63 pM (95% confidence interval: 7 to 238 pM). This compares well with the experimentally measured ranges for different seed-matched targets: 4 to 210 pM for fly, 13 to 26 pM for mouse (15).

A histogram of the results for all the seed sequences in the dataset is shown in Fig. S2A. The distribution has 8 peaks, roughly corresponding to the fact that the seed-target complex can have between 0 and 7 CG pairs. The more CG pairs relative to AU pairs, the stronger the binding and the lower the $K_{\rm D}$. To model the effect of having a 6 nt seed, in Fig. S2B the calculation was redone after randomly deleting 1 nucleotide from each original seed. Similarly Fig. S2C shows the results for 5 nt seeds, after randomly deleting 2 nucleotides per seed. As expected, shorter seeds lead to overall weaker binding, shifting the distribution to larger $K_{\rm D}$ values.

S.V. Analysis of experimental microRNA systems

In this section we use our theoretical framework to analyze data from two earlier experimental works on microRNA-mediated noise regulation. As mentioned in Sec. S.I., the basic mathematical framework for miRNA-regulated protein production, which is the starting point for our theory, has already been validated in Ref. (2) in a variety of experimental scenarios. Here we show another aspect of the analysis, interpreting the experimental data via the optimal noise filter formulation of the theory. We focus on two systems, both involving endogenous miRNAs in living cells interacting with binding sites on 3'UTR regions from endogenous genes, with the latter coupled to fluorescent reporters that allow direct measurements of regulation strength and noise reduction. The genes are: i) *Lats2* in mouse embryonic stem cells (mESCs) (2); ii) *sens* in *Drosophila* wing disc cells (16).

Let us start with an overview of the analysis procedure, before turning to specific details for the two systems. In each experiment there is a comparison between a miRNA-regulated system, and one without regulation, where the 3'UTR target sites are modified to prevent miRNA binding. This allows us to extract the error E from the relative noise levels in the regulated versus unregulated case, as defined in Eq. [S12], as well as the regulation strength R. The experimental measurements are carried out over a population of cells where the protein expression levels (as quantified by the fluorescent reporter) vary, and we will use X to denote the measure of protein levels in a cell, with X corresponding to fluorescence intensity (in arbitrary units) in the *Lats2* case, and to protein numbers in the *sens* case (where the calibration between intensity and copy numbers was carried out). The noise filter characteristics in a given cell will depend on $X \propto \bar{p} \propto \bar{m}$, and hence the experimental values of $E^{\rm exp}(X)$ and $R^{\rm exp}(X)$ will both vary with X. In our analysis, we fit $E^{\rm exp}(X)$ to Eq. [S14], our analytical result in the limit where the protein degradation rate constant is much smaller than the mRNA degradation rate coefficient ($\phi \ll 1$). Plugging Eq. [S7] and $R^{\rm exp}(X)$ into Eq. [S14], we get the following theoretical expression relating the two measured quantities:

$$E^{\rm exp}(X) = \frac{\left(1 - R^{\rm exp}(X)\right)\left(1 + \frac{R^{\rm exp}(X)}{\gamma_\mu}\left(\frac{1}{\widehat{K}_{\rm M}/X+1}\right)\right)}{\left(1 - R^{\rm exp}(X)\left(\frac{1}{\widehat{K}_{\rm M}/X+1}\right)\right)^2}. \qquad \text{[S38]}$$

where we define a rescaled Michaelis-Menten constant $\widehat{K}_{\rm M} = K_{\rm M} X/\bar{m}$, with $X/\bar{m}$ being the constant scaling factor between X and mRNA number $\bar{m}$. Eq. [S38] has only two unknowns, γ_μ and $\widehat{K}_{\rm M}$, which can be estimated via fitting to the experimental data for $E^{\rm exp}(X)$ and $R^{\rm exp}(X)$ as a function of X. Once $\widehat{K}_{\rm M}$ is determined from the fit, we can use main text Eq. [4] for $K^*_{\rm M}$ to find the ratio of actual to optimal Michaelis-Menten constants, $K_{\rm M}/K^*_{\rm M} = \widehat{K}_{\rm M}\gamma_\mu\sigma_\epsilon^{1/2}/X$, which is plotted in main text Fig. 4. Note that this ratio scales inversely with expression level, $K_{\rm M}/K^*_{\rm M} \propto 1/X$, and thus cells with different X will be closer or further away from optimality. As discussed in the main text, the results demonstrate that across a physiological range of protein expression levels, both experimental systems exhibit $K_{\rm M}$ roughly within an order of magnitude of $K^*_{\rm M}$.

miRNA regulation of *Lats2* in mouse embryonic stem cells (2): The regulation strength R is obtained by the relative expression levels in regulated and unregulated cases, i.e., $R = 1 - \bar{p}_R/\bar{p}_0$. This was determined via a dual reporter system and is almost constant at $R^{\rm exp}(X) \approx 0.8$ for all expression levels X (Fig. S3A), quantified in terms of the fluorescent marker (mCherry) intensity X. The protein expression noise in these experiments was reported in terms of the coefficient of variation,

$$\eta_\lambda = \sqrt{\frac{\langle \delta p^2 \rangle_\lambda}{\bar{p}^2}} \qquad \text{[S39]}$$

where the subscript $\lambda = 0, R$ applies to unregulated and regulated cases respectively. These are shown in Fig. S3B as blue (regulated data), black (unregulated data). Our theory can be related to the experimental data by estimating the error value,

$$E = \frac{F_R - 1}{F_0 - 1} \approx \frac{F_R}{F_0} \qquad \text{[S40]}$$

where $F_\lambda = \langle \delta p^2 \rangle_\lambda / \bar{p}$ for $\lambda = 0, R$ (regulated and unregulated) and the approximation holds when $F_R, F_0 \gg 1$, which is generally true for protein expression (17). Thus we can estimate the experimental error $E^{\rm exp}(X)$ from the reported data via

$$E^{\rm exp}(X) = \frac{\eta_R^2(X)}{\eta_0^2(X)}, \qquad \text{[S41]}$$

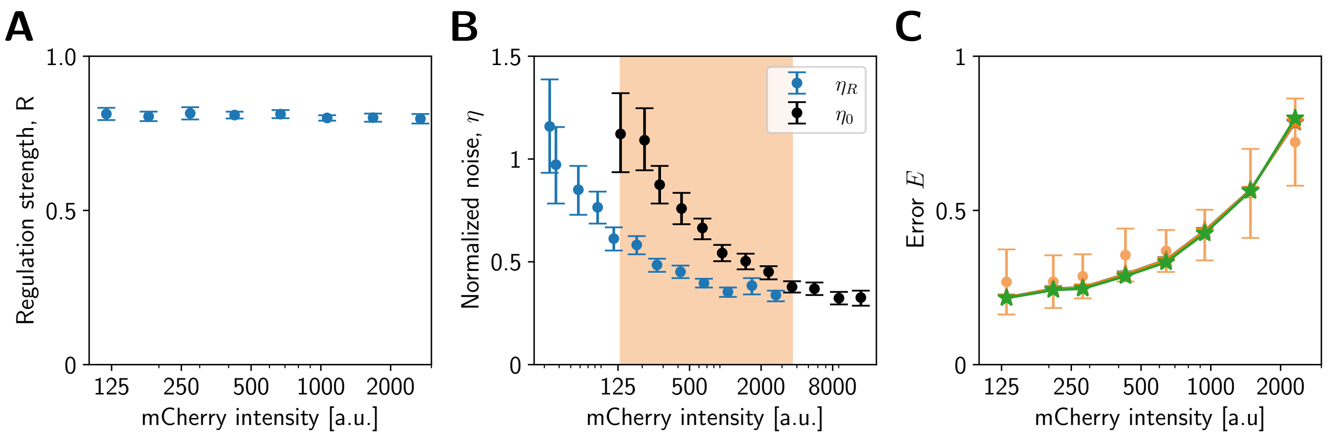

Fig. S3. Analysis of the experimental mCherry expression from the $Lats2$-microRNA system (2). (A) Experimentally determined regulation strengths R with varying mCherry intensity. (B) Experimental data for noise levels in unregulated (η_0, black) and regulated (η_R, blue) mCherry expression. (C) We span mCherry intensity levels, allowing a direct comparison (within the orange shaded region of (B)) of the noise levels, and transform them to error E (orange) using Eq.[S41] and numerical interpolation. The solid lines (multi-color) with stars as points display best-fit curves for $\gamma_\mu = 0.001, 0.01, 0.114$. These three curves closely overlap, so are indistinguishable.

through which we obtain $E^{\rm exp}(X)$ as a function of X in the overlapping region (shaded region in Fig. S3B). Since $\eta_R(X)$ and $\eta_0(X)$ are not measured at exactly the same X values, we use interpolation to match the values, and show the results for the error $E^{\rm exp}(X)$ in Fig. S3C (orange points). To calculate the error bars, we have used the appropriate mean and standard deviation error propagation rules from the original data.

To apply Eq. [S38], we use the average $R^{\rm exp}(X)$ value and perform a chi-squared fitting to $E^{\rm exp}(X)$ using its mean and standard deviations. The quality of fitting is relatively insensitive to $\gamma_\mu = d_\mu/d_{m\mu}$. Since it is assumed γ_μ should be smaller than 1 (degradation of the miRNA-mRNA complex is faster than miRNA alone), but we do not have good literature estimates for $d_{m\mu}$, we take a biologically plausible range of possible γ_μ values $\gamma_\mu = 0.001 - 0.114$. The results are shown in Fig. S3C, and curves for different γ_μ values are essentially indistinguishable, all providing good fits to the experimental data.

In Fig. 4A of the main text, we plot the inferred Michaelis-Menten constants normalized by the optimal Michaelis-Menten value, $K_{\rm M}/K_{\rm M}^* = \widehat{K}_{\rm M}(\gamma_\mu)\gamma_\mu\sigma_\epsilon^{1/2}/X$, where σ_ϵ is the ratio of metabolic costs of microRNA and mRNA transcripts. The points represent fits using the Table S1 values of γ_μ and σ_ϵ, and the colored regions show the uncertainty due to possible ranges of γ_μ and σ_ϵ. The ratio $K_{\rm M}/K_{\rm M}^*$ depends very weakly on the choice of γ_μ, so the range $\gamma_\mu = 0.001 - 0.114$ does not significantly affect the estimate (in fact even extending the range to $\gamma_\mu < 0.001$ leaves the uncertainty region for $K_{\rm M}/K_{\rm M}^*$ essentially unchanged). For σ_ϵ we chose the range $\sigma_\epsilon = 0.0014 - 0.5$ as follows. The energetic costs of miRNA and mRNA transcription were introduced in Sec. S.III, with $\sigma_\epsilon = L_{\mu,\rm pri}/L_{\rm m,pre}$. The average estimated lengths of pri-miRNA $L_{\mu,\rm pri} = 2,500$ and pre-mRNAs $L_{\rm m,pre} = 20,000$ lead to $\sigma_\epsilon = 0.125$ (Table S1). We can narrow our search knowing that $L_{\rm m,pre} \approx 50,000$ for the Lats2 gene (18). However, the pri-miRNA transcripts are largely unknown due to fast transcription from primary to precursor structure. Moreover, a single pri-miRNA can lead to multiple copies of pre-miRNA. Thus, despite proposed methods (19, 20), we lack this information for mouse microRNAs. Nevertheless, considering the lengths of pre-miRNAs, which are about 70 nucleotides (21), gives an approximate lower bound: $L_{\mu,\rm pri} \geq L_{\mu,\rm pre} = 70$, which yields to $\sigma_\epsilon = 0.0014$ (note that this is a strict underestimation). Since the energetic costs of assembling miRNA are expected to be smaller than mRNA, we use $\sigma_\epsilon = 0.5$ as an upper bound. The lowest and highest $K_{\rm M}/K_{\rm M}^*$ values (boundaries of colored region in Fig. 4A) correspond to the results for lowest and highest values of both γ_μ and σ_ϵ.

Finally, as a consistency check, we can attempt to approximately convert the value of the inferred $K_{\rm M}$ into units of molar concentration. For this, we need a conversion factor between mCherry intensities and mRNA concentration. In Fig. 4B of Ref. (2), the probability density of mRNA levels in the mESC transcriptome is correlated with the range of mCherry fluorescence intensities. The peak value (most likely mRNA level in RPKM units) corresponds to intensity $X^\dagger = 510$. While we do not know the peak value for mESC cells in terms of copy numbers, we can use data from mouse fibroblasts (6) as a rough proxy. There the peak occurs around a copy number $\bar{m}^\dagger = 8$ (the most likely number of mRNAs from a single gene). Assuming a cell volume of 2,000 μm^3, this amounts to molar density $[m]^\dagger_{\rm fibroblast} = 0.0066$ nM. If we take mESC concentration at the peak $[m]^\dagger_{\rm mESC}$ to be similar, then the conversion factor is $[m]^\dagger_{\rm mESC}/X^\dagger = 1.29 \times 10^{-5}$nM/mCherry intensity. We can obtain $K_{\rm M}$ in terms of molar concentration using $K_{\rm M} = \widehat{K}_M[m]/X$ and the conversion factor, which yields $K_{\rm M} = 8.35, 0.86, 0.1$ nM respectively for the possible parameter values $\gamma_\mu = 0.001, 0.01, 0.114$. For comparison, $K_{\rm M}$ for a miRNA system in mouse fibroblasts was measured to be 0.1 nM (15), so our estimated $K_{\rm M}$ numbers seem plausible (particularly at $\gamma_\mu = 0.114$, the value from Table S1 which we used for most of our calculations).

miRNA regulation of *sens* in *Drosophila* wing disc cells (16): These experiments use a similar technique to the one described above, with reporter proteins attached to the *sens* 3'UTR used to investigate the noise regulation mediated by

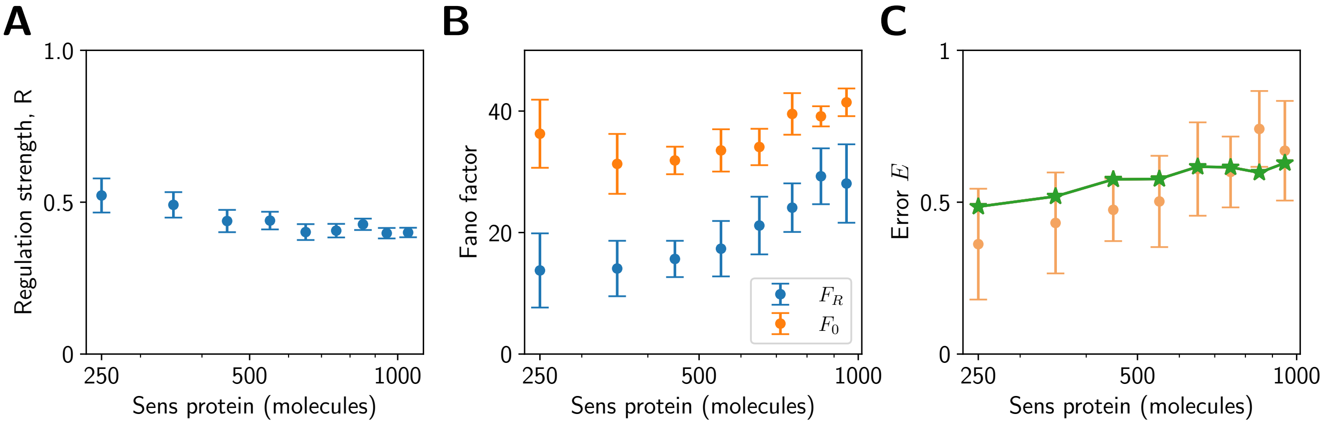

Fig. S4. Analysis of the experimental *sens*-miRNA system in *Drosophila* (16). (A) Experimentally determined regulation strengths R with varying Sens protein numbers. (B) Experimental data for the Fano factors of the unregulated (F_0, orange) and regulated (F_R, blue) cases. (C) Experimental results for the error E (orange), compared to best-fit theoretical curves (multi-color lines with stars correspond to $\gamma_\mu = 0.001, 0.01, 0.114$, and appear indistinguishable).

miRNAs. Comparison of noise levels is made between proteins having 3'UTR sequences with and without miR-9a binding sites (mCherry Sens wild type and mCherry Sens mutant). We obtained the regulation strengths $R^{\text{exp}}(X)$ by using the raw data for relative expression levels of mCherry Sens wild type and sfGFP Sens mutant proteins. This is plotted in Fig. S4A. The protein expression noise levels are reported in terms of Fano factors F_0 and F_R, and we binned the data along different expression levels (Fig. S4B). E^{exp} can then be directly obtained from:

$$E^{\text{exp}} = \frac{F_R - 1}{F_0 - 1}, \quad \text{[S42]}$$

which is shown in Fig. S4C. We then apply a fitting procedure identical to the one described in the *Lats2* example.

Main text Fig. 4B plots the inferred ratio $K_{\text{M}}/K_{\text{M}}^* = \widehat{K}_{\text{M}}(\gamma_\mu)\gamma_\mu\sigma_\epsilon^{1/2}/X$, with points representing fits using the parameter values from Table S1. The colored uncertainty region corresponds to varying $\gamma_\mu = 0.001 - 0.114$ and $\sigma_\epsilon = 0.014 - 0.5$. In this case, the lower bound for σ_ϵ is obtained using the pre-mRNA length of the *sens* gene $L_{\text{m,pre}} \approx 5,000$ (22) and $L_{\mu,\text{pri}} \geq L_{\mu,\text{pre}} = 70$, yielding $\sigma_\epsilon = 0.014$.

References

1. DT Gillespie, The chemical langevin equation. *J. Chem. Phys.* **113**, 297–306 (2000).
2. JM Schmiedel, et al., Microrna control of protein expression noise. *Science* **348**, 128–132 (2015).
3. S Mukherji, et al., Micrornas can generate thresholds in target gene expression. *Nat. Genet.* **43**, 854–859 (2011).
4. M Lynch, GK Marinov, The bioenergetic costs of a gene. *Proc. Natl. Acad. Sci.* **112**, 15690–15695 (2015).
5. D Hathcock, J Sheehy, C Weisenberger, E Ilker, M Hinczewski, Noise filtering and prediction in biological signaling networks. *IEEE Trans. Mol. Biol. Multi-Scale Commun.* **2**, 16–30 (2016).
6. B Schwanhäusser, et al., Global quantification of mammalian gene expression control. *Nature* **473**, 337–342 (2011).
7. MJ Marzi, et al., Degradation dynamics of micrornas revealed by a novel pulse-chase approach. *Genome Res.* **26**, 554–565 (2016).
8. ER Kingston, DP Bartel, Global analyses of the dynamics of mammalian microrna metabolism. *Genome Res.* **29**, 1777–1790 (2019).
9. B Alberts, et al., *Molecular Biology of the Cell.* (Garland Science), (2015).
10. G Song, L Wang, Mir-433 and mir-127 arise from independent overlapping primary transcripts encoded by the mir-433-127 locus. *PloS One* **3**, e3574 (2008).
11. HK Saini, AJ Enright, S Griffiths-Jones, Annotation of mammalian primary micrornas. *BMC Genom.* **9**, 1–19 (2008).
12. R Milo, P Jorgensen, U Moran, G Weber, M Springer, Bionumbers—the database of key numbers in molecular and cell biology. *Nucleic Acids Res.* **38**, D750–D753 (2010).
13. R Lorenz, et al., Viennarna package 2.0. *Algorithms Mol. Biol.* **6**, 1–14 (2011).
14. V Agarwal, GW Bell, JW Nam, DP Bartel, Predicting effective microrna target sites in mammalian mrnas. *eLife* **4**, e05005 (2015).
15. LM Wee, CF Flores-Jasso, WE Salomon, PD Zamore, Argonaute divides its rna guide into domains with distinct functions and rna-binding properties. *Cell* **151**, 1055–1067 (2012).
16. R Giri, et al., Ordered patterning of the sensory system is susceptible to stochastic features of gene expression. *eLife* **9**, e53638 (2020).

17. EM Ozbudak, M Thattai, I Kurtser, AD Grossman, A Van Oudenaarden, Regulation of noise in the expression of a single gene. *Nat. Genet.* **31**, 69–73 (2002).
18. JA Blake, et al., Mouse genome database (mgd): Knowledgebase for mouse–human comparative biology. *Nucl. Acids Res.* **49**, D981–D987 (2021).
19. J Qian, et al., The full-length transcripts and promoter analysis of intergenic micrornas in drosophila melanogaster. *Genomics* **97**, 294–303 (2011).
20. K Bedi, MT Paulsen, TE Wilson, M Ljungman, Characterization of novel primary mirna transcription units in human cells using bru-seq nascent rna sequencing. *NAR Genom. Bioinform.* **2**, lqz014 (2020).
21. Y Lee, K Jeon, JT Lee, S Kim, VN Kim, Microrna maturation: stepwise processing and subcellular localization. *The EMBO journal* **21**, 4663–4670 (2002).
22. LS Gramates, et al., Flybase: a guided tour of highlighted features. *Genetics* **220**, iyac035 (2022).
